# The alarming problems of confounding equivalence using logistic regression models in the perspective of causal diagrams

**DOI:** 10.1186/s12874-017-0449-7

**Published:** 2017-12-28

**Authors:** Yuanyuan Yu, Hongkai Li, Xiaoru Sun, Ping Su, Tingting Wang, Yi Liu, Zhongshang Yuan, Yanxun Liu, Fuzhong Xue

**Affiliations:** 1 Department of Biostatistics, School of Public Health, Shandong University, Jinan, People’s Republic of China; 2 School of Mathematical Sciences, Peking University, Beijing, People’s Republic of China; 3 Cheeloo Research Center for Biomedical Big Data, Shandong University, Jinan, People’s Republic of China

**Keywords:** Confounding equivalence, Logistic regression model, Inverse probability weighting based marginal structural model, Simulation study, Causal diagrams

## Abstract

**Background:**

Confounders can produce spurious associations between exposure and outcome in observational studies. For majority of epidemiologists, adjusting for confounders using logistic regression model is their habitual method, though it has some problems in accuracy and precision. It is, therefore, important to highlight the problems of logistic regression and search the alternative method.

**Methods:**

Four causal diagram models were defined to summarize confounding equivalence. Both theoretical proofs and simulation studies were performed to verify whether conditioning on different confounding equivalence sets had the same bias-reducing potential and then to select the optimum adjusting strategy, in which logistic regression model and inverse probability weighting based marginal structural model (IPW-based-MSM) were compared. The “*do*-calculus” was used to calculate the true causal effect of exposure on outcome, then the bias and standard error were used to evaluate the performances of different strategies.

**Results:**

Adjusting for different sets of confounding equivalence, as judged by identical Markov boundaries, produced different bias-reducing potential in the logistic regression model. For the sets satisfied G-admissibility, adjusting for the set including all the confounders reduced the equivalent bias to the one containing the parent nodes of the outcome, while the bias after adjusting for the parent nodes of exposure was not equivalent to them. In addition, all causal effect estimations through logistic regression were biased, although the estimation after adjusting for the parent nodes of exposure was nearest to the true causal effect. However, conditioning on different confounding equivalence sets had the same bias-reducing potential under IPW-based-MSM. Compared with logistic regression, the IPW-based-MSM could obtain unbiased causal effect estimation when the adjusted confounders satisfied G-admissibility and the optimal strategy was to adjust for the parent nodes of outcome, which obtained the highest precision.

**Conclusions:**

All adjustment strategies through logistic regression were biased for causal effect estimation, while IPW-based-MSM could always obtain unbiased estimation when the adjusted set satisfied G-admissibility. Thus, IPW-based-MSM was recommended to adjust for confounders set.

**Electronic supplementary material:**

The online version of this article (10.1186/s12874-017-0449-7) contains supplementary material, which is available to authorized users.

## Background

Causal inference is a key task in epidemiology which discovers the causality between exposure and outcome. Theoretically, causality is the difference in outcome caused by a change in exposure, which can be gotten by ‘*do-calculus*’ in observational studies [1]. In practice, however, as exposure is impossible to intervene in analytic epidemiology, confounders inevitably distort the causal effect of exposure on outcome [2–5]. For majority of epidemiologists, adjusting for confounders using logistic regression model for dichotomous outcomes is the routine method [6–10]. Although some studies have verified that different adjustment strategies in logistic regression models might lead to different magnitudes of bias (the difference of the estimation minus the true causal effect) and precision [8, 11], it is still the most commonly used strategy in analytic epidemiologic studies. This phenomenon is mainly attributed to their vague knowledge about the behaviour of logistic regression model. For causal inference in observational study, the inverse probability weighting based marginal structural model (IPW-based-MSM) has been confirmed as an unbiased causal effect estimation approach to adjust for measured confounders [12–14]. Unfortunately, the advantages of IPW-based-MSM are not recognized by most epidemiologists. Furthermore, for both logistic regression and IPW-based-MSM, the selection of adjustment variables sets remains a big challenge. Fortunately, the concept of confounding equivalence (*c-equivalence*) proposed by Judea Pearl might help us to select adjusting strategies [15].

The *c-equivalence* is presented to determine whether two variables sets are equally valuable for adjustment, namely, whether adjustment for one set is guaranteed to have the same asymptotic bias as adjustment for the others [15]. Tests for *c-equivalence* are fairly easy to perform through a necessary and sufficient condition [15, 16], and they can also be implemented by propensity score methods [17]. This provides us a strategy for selecting adjusting variables sets when using logistic regression models and IPW-based-MSMs, which help to clarify whether adjusting for different *c-equivalent* sets has same bias-reducing potential.

**Fig. 1:**
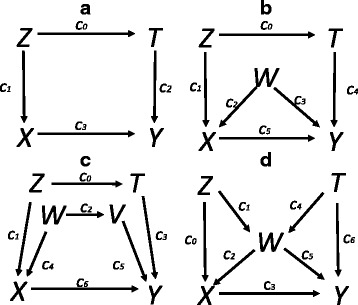
Four typical causal diagrams with various confounding paths from simple to complex for the target causal path *X*→*Y*. **a** contains only one confounding path (*X*←*Z*→*T*→*Y*). **b** contains two confounding paths (*X*←*Z*→*T*→*Y*, *X*←*W*→*Y*). Two confounding paths (*X*←*Z*→*T*→*Y*, *X*←*W*→*V*→*Y*) that have another node (*V*) are included in (**c**). **d** has three confounding paths (*X*←*W*→*Y*, *X*←*Z*→*W*→*Y* and *X*←*W*←*T*→*Y*). *X* and *Y* indicates exposure and outcome respectively. *T*, *Z*, *W* and *V* are all confounders that can be observed. {*c*_0_, *c*_1_, *c*_2_, *c*_3_, *c*_4_, *c*_5_} are the effect parameters. For example, the effect of *Z* on *T* is *c*_0_

In this paper, we focused on 4 typical causal diagrams (Fig. 1), which summarized the generalization of *c-equivalence* to detect the performances of logistic regression models and IPW-based-MSMs under the framework of *c-equivalence*. Both theoretical proofs and simulation studies were performed to determine whether adjusting for the sets of *c-equivalence* had the same bias-reducing potential and observed their precision in logistic regression models and IPW-based-MSMs respectively, and further comparing the performances of *c-equivalence* between these two models through assessing their accuracy (bias) and precision (standard error). Our aim was to highlight the problems of *c-equivalence* using logistic regression model as well as the advantages of IPW-based-MSM.

## Methods

### *C-equivalence* and its test

Let *X*, *Y* and *Z* be three disjoint subsets of discrete variables, and *P*(*x*, *y*, *z*) are their joint distribution. The causal effect of *X* on *Y* can be defined as $P\left(y| do(x)\right)=\sum \limits_zP\left(y|x,z\right)p(z)$ [5, 18, 19], where a sufficient set *Z* is chosen to include variables judged as “confounders” [16, 20, 21]. In this framework, the two confounders sets *T* and *Z* are *c-equivalent* if $\sum \limits_tP\left(y|x,t\right)P(t)=\sum \limits_zP\left(y|x,z\right)P(z)$ ∀*x*, *y*. This means that adjustment for set *T* or *Z* would produce the same asymptotic bias relative to the target causal effect quantity [15]. To meet the necessary and sufficient condition of *c-equivalence*, it is first necessary to define the G-admissibility of a variables set *S*, which satisfies the back-door criterion [19]: 1) No element of *S* is a descendant of *X*; 2) The elements of *S* block every path between *X* and *Y* that contains an arrow into *X*. Another condition of *c-equivalence* is the identical Markov boundary [15], which is defined as: let *S*_m_ be the minimal subset of *S* that satisfies the condition (*X* ⊥ *S*| *S*_*m*_)_*G*_. This means that measurement of *S*_m_ renders *X* independent of all other members of *S*, and no proper subset of *S*_m_ has this property. Therefore, the necessary and sufficient conditions for *T* and *Z* to be *c-equivalent* are that at least one of the following conditions hold: 1) *T*_m_ = *Z*_m_; and 2) *T* and *Z* are *G*-admissible [15].

As an example, Fig. 1 illustrates the four typical causal diagrams with simple and complex confounding paths for the target causal path *X*→*Y* [22]. For instance, Fig. 1d contains three confounding paths *X*←*W*→*Y*, *X*←*Z*→*W*→*Y* and *X*←*W*←*T*→*Y*, with three corresponding confounders *Z*, *W* and *T* [22, 23]. Theoretically, conditioning on {*Z, W*}, {*T, W*} or {*Z, T, W*} can achieve the same bias-reducing potential [23]. Thus, they are *c-equivalent*.

### Statistical methods for adjusting for confounders

Expect for the well-known logistic regression model which is the habitual method for most of epidemiologists, IPW-based-MSM is an alternative approach that can obtain the unbiased causal effect estimation [24, 25]. In IPW-based-MSM, the unbiased causal effect is estimated by inverse probability weighted which can correct for confounding bias [26]. In this paper, the following stabilized weights, which has been recommended to increase the statistical efficiency and to achieve better coverage of the confidence intervals, were used [13, 27],

$$ {sw}_i=\frac{P\left(X={x}_i\right)}{P\left(X={x}_i|{Z}_i={z}_i\right)} $$

where *Z* is a set of variables which are considered to be confounders. By weighting the original observations using the stabilized weights (*sw*_*i*_), we can fit the following marginal structural model to estimate the causal effect of *X* on *Y*,

$$ \mathrm{logit}\kern0.1em P\left({Y}_x=1\right)={\beta}_0^{MSM}+{\beta}_1^{MSM}x $$

where the causal effect estimation of *X* on *Y* is ${\beta}_1^{MSM}$.

### Theoretical derivation for bias-reducing potential of *c-equivalence* under logistic regression model

Taking Fig. 1a as an example, we deduced whether adjusting for different *c-equivalence* sets had the same bias-reducing potential under logistic regression by the following procedure.

1) Based on the necessary and sufficient condition, *A*_1_ = {*Z*}, *A*_2_ = {*T*} and *A*_3_ = {*Z, T*} satisfied G-admissibility, thus they were equivalent, as denoted by *A*_1_ ≈ *A*_2_ ≈ *A*_3_.

2) Calculated the true causal effect (*ACE* ^log(*OR*)^) of *X* on *Y* through the average causal effect (*ACE*) on the scale of the logarithm odds ratio (OR),

$$ {ACE}^{\log (OR)}=\mathrm{logit}\left(P\left(Y=1| do\left(X=1\right)\right)\right)-\mathrm{logit}\left(P\left(Y=1| do\left(X=0\right)\right)\right) $$

3) Calculated the effect (${\beta}_X^{set}$) of *X* on *Y* by logistic regression,

$$ {\displaystyle \begin{array}{l}{\beta}_X^{A_1}=\mathrm{logit}\left(P\left(Y=1|X=1,{A}_1\right)\right)-\mathrm{logit}\left(P\left(Y=1|X=0,{A}_1\right)\right)\\ {}{\beta}_X^{A_2}=\mathrm{logit}\left(P\left(Y=1|X=1,{A}_2\right)\right)-\mathrm{logit}\left(P\left(Y=1|X=0,{A}_2\right)\right)\\ {}{\beta}_X^{A_3}=\mathrm{logit}\left(P\left(Y=1|X=1,{A}_3\right)\right)-\mathrm{logit}\left(P\left(Y=1|X=0,{A}_3\right)\right)\end{array}} $$

4) Calculated the biases${\beta}_X^{A_1}-{ACE}^{\log (OR)}$, ${\beta}_X^{A_2}-{ACE}^{\log (OR)}$ and ${\beta}_X^{A_3}-{ACE}^{\log (OR)}$, and then deduced whether ${\beta}_X^{A_1}-{ACE}^{\log (OR)}={\beta}_X^{A_2}-{ACE}^{\log (OR)}={\beta}_X^{A_3}-{ACE}^{\log (OR)}$.

### Simulation

Taking the four typical causal diagrams, which covered the generalization of *c-equivalence* (Fig. 1), as examples, a series of simulation studies were performed to determine whether adjusting for the sets of *c-equivalence* had the same bias-reducing potential and observed their precision in logistic regression models and IPW-based-MSMs respectively, further compared the performances of *c-equivalence* between these two models though assessing their accuracy and precision.

Four simulation scenarios were considered, and assumed that: 1) all variables were binary and followed a Bernoulli distributions; and 2) the effects of parent nodes on their child nodes were positive and log-linearly additive. Logistic regression models were used to simulate child nodes from their corresponding parent nodes.

For scenario 1 (Fig. 1a), the simulated data were generated as follows. Let*P*(*Z* = 1) = *π*. Then, *P*(*T* = 1| *Z*) = exp(*c*_0_ *Z* + *α*_1_)/(1 + exp(*c*_0_ *Z* + *α*_1_)) was used to derive the probability of child node *T* from its parent node *Z*. Similarly, *P*(*X* = 1| *Z*) = exp(*c*_1_ *Z* + *α*_2_)/(1 + exp(*c*_1_ *Z* + *α*_2_)) and *P*(*Y* = 1| *X*, *T*) = exp(*c*_3_ *X* + *c*_2_ *T* + *α*_0_)/(1 + exp(*c*_3_ *X* + *c*_2_ *T* + *α*_0_)) were used to obtain the probability of *X* = 1 and *Y* = 1, respectively, where the parameters *α*_0_, *α*_1_, *α*_2_ denoted the intercepts of *Y, T* and *X*, respectively, and each effect parameter (*c*_0_, *c*_1_, *c*_2_, *c*_3_) referred to the effect of the parent node on its corresponding child node. Simulated data was generated for 1000 subjects by above procedure.

In this scenario (Fig. 1a), variable sets *A*_1_ = {*Z*}, *A*_2_ = {*T*} and *A*_3_ = {*Z, T*} satisfied the necessary and sufficient conditions of *c-equivalence*; thus, *A*_1_ ≈ *A*_2_ ≈ *A*_3_. Therefore, we compared three adjustment strategies with the following six models,

model 1: $$ \mathrm{logit}\left(p\left(Y=1|X,{A}_1\right)\right)={{\widehat{\beta}}^{A_1}}_0+{\widehat{\beta}}_X^{A_1}X+{{\widehat{\beta}}^{A_1}}_Z\mathrm{Z} $$.

model 2: $$ \mathrm{logit}\left(p\left(Y=1|X,{A}_2\right)\right)={{\widehat{\beta}}^{A_2}}_0+{\widehat{\beta}}_X^{A_2}X+{{\widehat{\beta}}^{A_2}}_TT $$.

model 3: $$ \mathrm{logit}\left(p\left(Y=1|X,{A}_3\right)\right)={{\widehat{\beta}}^{A_3}}_0+{\widehat{\beta}}_X^{A_3}X+{{\widehat{\beta}}^{A_3}}_TT+{{\widehat{\beta}}^{A_3}}_ZZ $$.

model 4: $$ \mathrm{logit}\kern0.1em P\left({Y}_x^{A_1}=1\right)={\widehat{\beta}}_0^{MSM\_{A}_1}+{\widehat{\beta}}_x^{MSM\_{A}_1}x $$ $$ {sw}_i^{A_1}=\frac{P\left(X={x}_i\right)}{P\left(X={x}_i|{A}_{1i}={A}_{1i}\right)} $$.

model 5: $$ \mathrm{logit}\kern0.1em P\left({Y}_x^{A_2}=1\right)={\widehat{\beta}}_0^{MSM\_{A}_2}+{\widehat{\beta}}_x^{MSM\_{A}_2}x $$ $$ {sw}_i^{A_2}=\frac{P\left(X={x}_i\right)}{P\left(X={x}_i|{A}_{2i}={A}_{2i}\right)} $$.

model 6: $$ \mathrm{logit}\kern0.1em P\left({Y}_x^{A_3}=1\right)={\widehat{\beta}}_0^{MSM\_{A}_3}+{\widehat{\beta}}_x^{MSM\_{A}_3}x $$ $$ {sw}_i^{A_3}=\frac{P\left(X={x}_i\right)}{P\left(X={x}_i|{A}_{3i}={A}_{3i}\right)} $$.

where${\widehat{\beta}}_X^{A_1}$, ${\widehat{\beta}}_X^{A_2}$, ${\widehat{\beta}}_X^{A_3}$, ${\widehat{\beta}}_X^{MSM\_{A}_1}$, ${\widehat{\beta}}_X^{MSM\_{A}_2}$ and ${\widehat{\beta}}_X^{MSM\_{A}_3}$ denoted the causal effect estimations after conditioning on *A*_1_ *, A*_2_ and *A*_3_ by logistic regression and IPW-based-MSM, respectively. Given the true causal effect $A\widehat{C}{E}^{\log (OR)}$ calculated by *do-calculus*, both the biases (${\widehat{\beta}}_X^{A_1}-A\widehat{C}{E}^{\log (OR)}$,${\widehat{\beta}}_X^{A_2}-A\widehat{C}{E}^{\log (OR)}$, ${\widehat{\beta}}_X^{A_3}-A\widehat{C}{E}^{\log (OR)}$, ${\widehat{\beta}}_x^{MSM\_{A}_1}-A\widehat{C}{E}^{\log (OR)}$, ${\widehat{\beta}}_x^{MSM\_{A}_2}-A\widehat{C}{E}^{\log (OR)}$, ${\widehat{\beta}}_x^{MSM\_{A}_3}-A\widehat{C}{E}^{\log (OR)}$) and their corresponding standard errors ($\mathrm{SE}\left({\widehat{\beta}}_X^{A_1}\right)$, $\mathrm{SE}\left({\widehat{\beta}}_X^{A_2}\right)$, $\mathrm{SE}\left({\widehat{\beta}}_X^{A_3}\right)$, $\mathrm{SE}\left({\widehat{\beta}}_X^{MSM\_{A}_1}\right)$, $\mathrm{SE}\left({\widehat{\beta}}_X^{MSM\_{A}_2}\right)$ and $\mathrm{SE}\left({\widehat{\beta}}_X^{MSM\_{A}_3}\right)$) were used to identify whether adjusting for different *c-equivalence* sets *A*_1_, *A*_2_ or *A*_3_ still produced the same bias-reducing under the logistic regression model and IPW-based-MSM, further to evaluate their accuracy and precision.

For scenario 2 (Fig. 1b), similar simulation data sets were created as scenario 1. In this scenario, *A*_1_ = {*Z, W*}, *A*_2_ = {*T, W*} and *A*_3_ = {*Z, T, W*} satisfied G-admissibility; thus, *A*_1_ ≈ *A*_2_ ≈ *A*_3_. Therefore, three corresponding logistic regression models and three corresponding IPW-based-MSMs conditional on *A*_1_ *, A*_2_ or *A*_3_ were constructed to identify whether the *c-equivalence* has identical biases and to evaluate their precisions. In addition, *B*_1_ = {*Z*} was *c-equivalent* to *B*_2_ = {*Z, T*}, namely, *B*_1_ ≈ *B*_2_, due to their identical Markov boundary, written as *B*_1m_ = *B*_2m_ = {*Z*}. Therefore, four corresponding models conditioning on *B*_1_ or *B*_2_ were used to calculate the biases and standard errors.

In scenario 3 (Fig. 1c), the simulated data was generated in the same way as in scenario 1. In addition, the sets *A*_1_ = {*Z*} ≈ *A*_*2*_ = {*Z*, *T*} and *B*_1_ = {*W*} ≈ *B*_2_ = {*W,V*} were separately *c-equivalent* due to *A*_1m_ = *A*_2m_ = {*Z*} and *B*_1m_ = *B*_2m_ = {*W*}. As *A*_1_ ≈ *A*_*2*_ and *B*_1_ ≈ *B*_2_ were identical in the *c-equivalence* mechanism, it was sufficient to analyze one group to explore the *c-equivalence* mechanism of the identical Markov boundary. Thus, we constructed two logistic regression models and two IPW-based-MSMs conditioning on *A*_1_ or *A*_2_ to explore their *c-equivalence* and to evaluate their precision. Furthermore, as variables sets *C*_1_ = {*Z*,*W*}, *C*_2_ = {*T*,*V*} and *C*_3_ = {*Z*,*W*,*T*,*V*} blocked all back-door paths from *X* to *Y*, they were admissible and equivalent, *C*_1_ ≈ *C*_2_ ≈ *C*_3_. Therefore, the six corresponding models conditional on *C*_1_, *C*_2_ or *C*_3_ were performed to identify biases and precisions.

For scenario 4 (Fig. 1d), following the path directions, simulation data sets were created same with scenario 1. *A*_1_ = {*Z, W*}, *A*_2_ = {*T, W*} and *A*_3_ = {*Z, T, W*} satisfied G-admissibility; thus, *A*_1_ ≈ *A*_2_ ≈ *A*_3_. Their corresponding three logistic regression models and three IPW-based-MSMs conditional on *A*_1_ *, A*_2_ or *A*_3_ were used to observe the biases and precisions.

For each of the 4 simulation scenarios, we varied across the effect of a specific edge given the others fixed with 1000 simulation repetitions. The R (http://cran.r-project.org/) programming language was used to conduct the statistical simulations.

## Results

### Theoretical results for bias-reducing potential of *c-equivalence* under logistic regression model

Considered scenario 1 (Fig. 1a) as a typical diagram for deducing whether adjusting for different *c-equivalence* sets resulted in the same bias reduction under the logistic regression models. In this causal diagram, *A*_1_ = {*Z*}, *A*_2_ = {*T*} and *A*_3_ = {*Z, T*} composed the *c-equivalence* group, which satisfied the G-admissibility .

For *A*_1_ ≈ *A*_2_ ≈ *A*_3_ of *c-equivalence*, the true causal effect of *X* on *Y* was calculated as

$$ {\displaystyle \begin{array}{l}{ACE}^{\log (OR)}=\mathrm{logit}\left[P\Big(Y=1| do\left(X=1\right)\Big)\right]-\mathrm{logit}\left[P\Big(Y=1| do\left(X=0\right)\Big)\right]\\ {}{ACE}^{\log (OR)}=\mathrm{logit}\kern.2em \left[\sum \limits_{Z,T}P\left(Y=1|X=1,T\right)P\Big(T|Z\Big)P(Z)-\mathrm{logit}\left[\sum \limits_{Z,T}P\left(Y=1|X=0,T\right)P\Big(T|Z\Big)P(Z)\right.\right]\end{array}} $$

By conditioning on *A*_1_ = {*Z*}, the effect of *X* on *Y* was equal to

$$ {\displaystyle \begin{array}{l}{\beta}_{\mathrm{X}}^{A_1}=\mathrm{logit}\left[P\left(Y=1|X=1,Z\right)\right]-\mathrm{logit}\left[P\left(Y=1|X=0,Z\right)\right]\\ {}\kern1.7em =\mathrm{logit}\left[\sum \limits_TP\left(Y=1|X=1,T\right)P\left(T|Z\right)\right]-\mathrm{logit}\left[\sum \limits_TP\left(Y=1|X=0,T\right)P\left(T|Z\right)\right]\end{array}} $$

Similarly, the effect of *X* on *Y* when conditioning on *A*_2_ *=* {*T*} was equal to

$$ {\displaystyle \begin{array}{l}{\beta}_{\mathrm{X}}^{A_2}=\mathrm{logit}\left[P\left(Y=1|X=1,T\right)\right]-\mathrm{logit}\left[P\left(Y=1|X=0,T\right)\right]\\ {}\kern1.6em =\mathrm{logit}\left[P\left(Y=1|X=1,T\right)\sum \limits_TP\left(T|Z\right)\right]-\mathrm{logit}\left[P\left(Y=1|X=0,T\right)\sum \limits_TP\left(T|Z\right)\right]\end{array}} $$

Additionally, the effect of *X* on *Y* when conditioning on *A*_3_ = {*T*, *Z*} was equal to

$$ {\displaystyle \begin{array}{l}{\beta}_{\mathrm{X}}^{A_3}=\mathrm{logit}\left[P\left(Y=1|X=1,T,Z\right)\right]-\mathrm{logit}\left[P\left(Y=1|X=0,T,Z\right)\right]\\ {}\kern1.8em =\mathrm{logit}\left[P\left(Y=1|X=1,T\right)\right]-\mathrm{logit}\left[P\left(Y=1|X=0,T\right)\right]\end{array}} $$

After a series of derivations (Additional file 1: Appendix), we obtained ${\beta}_X^{A_2}={\beta}_X^{A_3}$ under any condition, suggesting that the bias-reducing after adjusting for *c-equivalence* sets *A*_*2*_ ≈ *A*_3_ was equivalent under the logistic regression model. ${\beta}_X^{A_1}={\beta}_X^{A_2}={\beta}_X^{A_3}$ only if *c*_2_ = 0  or *c*_3_ = 0, indicating that the bias-reducing after adjusting for *c-equivalence* sets *A*_1_ ≈ *A*_*2*_ ≈ *A*_3_, respectively, was equivalent in this situation. However, ${\beta}_X^{A_1}<{\beta}_X^{A_2}={\beta}_X^{A_3}$ if *c*_2_ ≠ 0 and *c*_3_ > 0, and ${\beta}_X^{A_1}>{\beta}_X^{A_2}={\beta}_X^{A_3}$ if *c*_2_ ≠ 0 and *c*_3_ < 0,which indicating an unequal bias-reducing after adjusting for *c-equivalence* sets *A*_1_ ≈ *A*_*2*_ ≈ *A*_3_ when both *c*_2_ and *c*_3_ were not equal to zero (for more details, see Appendix).

### Simulation results

#### Scenario 1

**Fig. 2:**
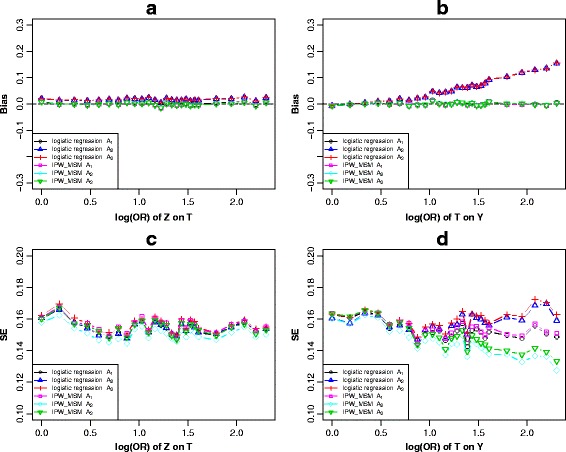
Scenario 1 (Fig. 1a), simulation results of the bias (**a** and **b**) and standard error (**c**) and (**d**) of *c-equivalence* sets *A*_1_ ≈ *A*_2_ ≈ *A*_3_ when varied across the log transformed odds ratio effect of *Z* on *T* and *T* on *Y*

For Fig. 1a, various simulation strategies were performed. From the panel a and panel b of Fig. 2 and Additional file 2: Figure S1, as for the logistic regression models, we observed that adjusting for the *c-equivalent* set *A*_2_ or *A*_3_ has resulted in approximate biases, but adjusting for set *A*_1_ was not equal to them. Moreover, the strategy of adjusting for *A*_1_ achieved the minimum bias. When adjusting for confounders by IPW-based-MSM, the estimations of all the strategies were approximate and unbiased. Panel c and d of Fig. 2 and Additional file 2: Figure S1 showed that adjusting for *A*_2_ by IPW-based-MSM achieved the highest precision in all situations. Thus, compared with logistic regression models, the IPW-based-MSM produced an unbiased causal effect estimation and the highest precision in this scenario. The optimal adjustment strategy was conditioning on *A*_2_. Although the estimations through logistic regression model were biased, adjusting for *A*_1_ produced a result nearest to the true causal effect.

When varying across the effect of *Z* on *T* with the other parameters fixed*,* the simulation results indicated that the biases of all six models (models 1–6) tended to be stable (Fig. 2a). Similar performances were observed when varying across the effect of *Z* on *X* (Additional file 2: Figure S1a). However, when varying across the effect of *T* on *Y* and keeping the other parameters constant, the bias showed a linear increasing trend after adjusting for set *A*_2_ or *A*_3_ under the logistic regression model, but was approximately to zero after adjusting for set *A*_1_. However, the biases remained stable under IPW-based-MSM (Fig. 2b). We observed similar trends with the effect of *X* on *Y* increasing (Additional file 2: Figure S1b).

#### Scenario 2

**Fig. 3:**
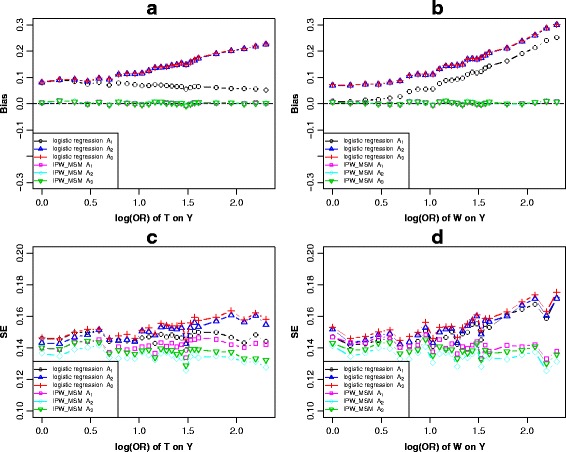
Scenario 2 (Fig. 1b), simulation results of the bias (**a** and **b**) and standard error (**c** and **d**) of *c-equivalence* sets *A*_1_ ≈ *A*_2_ ≈ *A*_3_ when varied across the log transformed odds ratio effect of *T* on *Y* and *W* on *Y*

In Fig. 1b, for the first *c-equivalent* subsets *A*_1_ = {*Z, W*}, *A*_2_ = {*T*, *W*} and *A*_3_ = {*Z, T, W*}, we observed that the bias after adjusting for set *A*_2_ was similar to that of *A*_3_ but not to that of *A*_1_, and the strategy of adjusting for *A*_1_ achieved the minimum bias under the logistic regression models, as shown in panels a and b of Fig. 3, Additional file 3: Figure S2 and Additional file 4: Figure S3 under logistic regression models. The adjustment of any confounding sets of *c-equivalent* subsets through IPW-based-MSM had the same bias-reducing potential and the estimations were unbiased. Panel c and d of these figures showed that adjusting for *A*_2_ under IPW-based-MSM achieved the highest precision in all situations. Thus, conditioning on any *c-equivalent* set that was satisfied G-admissibility through IPW-based-MSM produced an unbiased causal effect estimate and adjustment for *A*_2_ was the best strategy. When using logistic regression models to adjust for confounders, the optimal adjustment strategy was adjusting for variable subset *A*_1_.

In the logistic regression models, when keeping the other parameters constant, bias elevated with the effect of *T* on *Y* increasing when adjusting for *A*_2_ or *A*_3_, whereas it elevated in the opposite direction when adjusting for *A*_1_ (Fig. 3a). All three models revealed increased biases with the effects of *W* on *Y* increasing (Fig. 3b). Similar performances were observed when varying across the effect *X* on *Y* (Additional file 3: Figure S2b). When varying across the effect of *Z* on *T* with the other parameters fixed*,* the simulation results indicated that the biases of all three adjustment strategies tended to be stable (Additional file 3: Figure S2b). We observed similar trends with the increase of the effect of *Z* on *X* (Additional file 4: Figure S3a) or the effect of *W* on *X* (Additional file 4: Figure S3b). When adjusting for confounders through IPW-based-MSM, the biases of all three adjustment strategies tended to be stable in all situations.

**Fig. 4:**
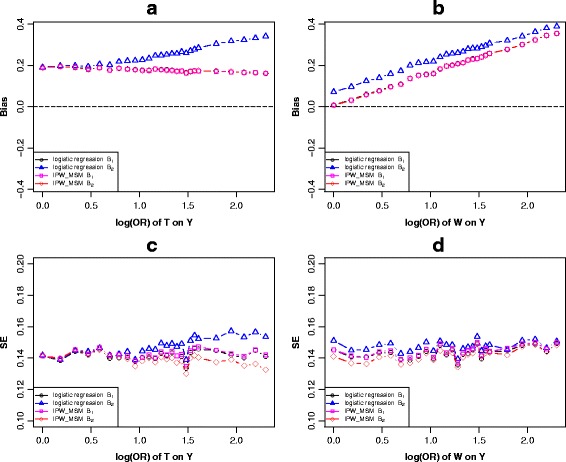
Scenario 2 (Fig. 1b), simulation results of the bias (**a** and **b**) and standard error (**c** and **d**) of *c-equivalence* sets *B*_1_ ≈ *B*_2_ when varied across the log transformed odds ratio effect of *T* on *Y* and *W* on *Y*

For another *c-equivalent* subsets *B*_1_ = {*Z*} and *B*_2_ = {*Z, T*}, panels a and b of Fig. 4, Additional file 5: Figure S4 and Additional file 6: Figure S5 showed that adjusting for *c-equivalence* set *B*_1_ or *B*_2_ had different bias-reducing, and the bias of adjusting for *B*_1_ was less than that of adjusting for *B*_2_ under the logistic regression models. For IPW-based-MSM, the biases were equivalent after adjusting for *B*_1_ or *B*_2_. Panels c and d of these figures showed that adjusting for *B*_2_ through IPW-based-MSM resulted in higher precision.

Keeping the other parameters constant, the bias elevated as the effect of *T* on *Y* increasing when adjusting for set *B*_2_, whereas it was stable after adjusting for *B*_1_ under logistic regression. A stable trend also appeared after adjusting for any sets through IPW-based-MSM (Fig. 4a). Similar performances were observed when varying across the effect of *X* on *Y* (Additional file 5: Figure S4b). When varying across the effect of *W* on *Y* with the other parameters fixed*,* the simulation results indicated that biases of four models revealed an increasing trend (Fig. 4b). Similar trends of the effect of *W* on *X* increasing were observed in Additional file 6: Figure S5b. When varying across the effect of *Z* on *T* with the other parameters fixed*,* the biases of the four models were stable (Additional file 5: Figure S4a). Similar performances were observed when varying across the effect of *Z* on *X* (Additional file 6: Figure S5a).

#### Scenario 3

**Fig. 5:**
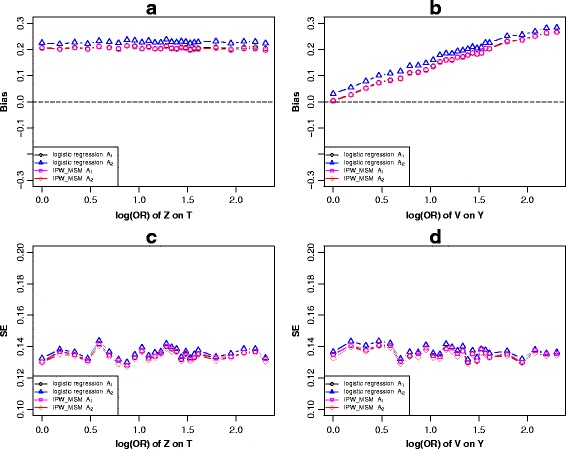
Scenario 3 (Fig. 1c), simulation results of the bias (**a** and **b**) and standard error (**c** and **d**) of *c-equivalence* sets *A*_1_ ≈ *A*_2_ when varied across the log transformed odds ratio effect of *Z* on *T* and *V* on *Y*

In Fig. 1c, for the first *c-equivalent* subsets, *A*_1_ = {*Z*} and *A*_*2*_ = {*Z*, *T*}, Fig. 5, Additional file 7: Figure S6 and Additional file 8: Figure S7 showed that adjusting for *c-equivalence* set *A*_1_ or *A*_2_ resulted in different bias-reducing, and the bias of adjusting for *A*_1_ was less than that after adjusting for *A*_2_ under logistic regression models. Then the biases were equal after conditioning on *A*_1_ and *A*_*2*_ via IPW-based-MSM. In consideration of the standard error, adjusting for *A*_2_ through IPW-based-MSM resulted in higher precision.

**Fig. 6:**
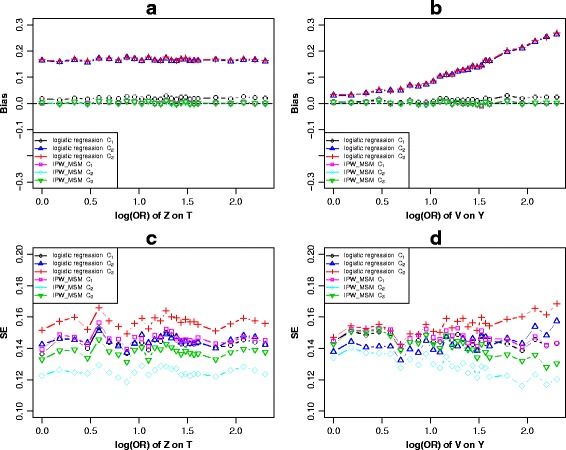
Scenario 3 (Fig. 1c), simulation results of the bias (**a** and **b**) and standard error (**c** and **d**) of *c-equivalence* sets *C*_1_ ≈ *C*_2_ ≈ *C*_3_when varied across the log transformed odds ratio effect of *Z* on *T* and *V* on *Y*

For other *c-equivalent* subsets *C*_1_ = {*Z*,*W*}, *C*_2_ = {*T*,*V*} and *C*_3_ = {*Z*,*W*,*T*,*V*}, the simulation result (Fig. 6, Additional file 9: Figure S8 and Additional file 10: Figure S9) showed that adjusting for the variable set *C*_2_ resulted in similar bias to that of set *C*_3_ but not to *C*_1_, and the strategy of adjusting for *C*_1_ resulted in the minimum bias under the logistic regression models. However, the estimations of all strategies conditioned by IPW-based-MSM were approximately equivalent and unbiased. For the standard error, conditioning on *C*_2_ by IPW-based-MSM resulted in the minimum standard error in all situations. Thus, IPW-based-MSM was a better method than logistic regression for controlling for confounders. The optimal adjustment strategy was conditioning on *C*_2_ by IPW-based-MSM in this scenario. Besides, adjusting for *A*_1_ produced the result that was nearest to the true causal effect under the logistic regression model.

#### Scenario 4

**Fig. 7:**
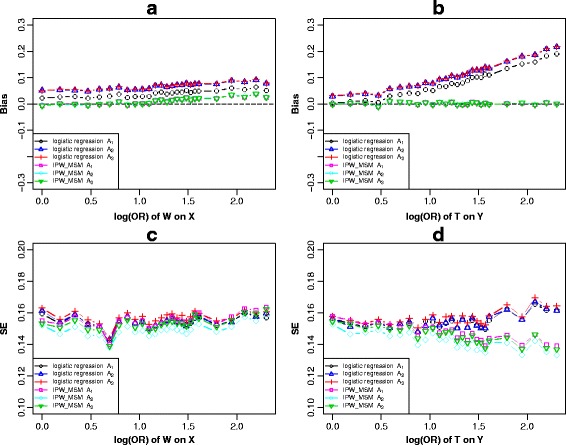
Scenario 4 (Fig. 1d), simulation results of the bias (**a** and **b**) and standard error (**c** and **d**) of *c-equivalence* sets *A*_1_ ≈ *A*_2_ ≈ *A*_3_when varied across the log transformed odds ratio effect of *W* on *X* and *T* on *Y*

For Fig. 1d, simulation results (Fig. 7, Additional file 11: Figure S10 and Additional file 12: Figure S11) showed that adjusting for *c-equivalence* set *A*_2_ or *A*_3_ had different bias-reducing but adjusting for *A*_1_ was not equal to them and the strategy of adjusting for *A*_1_ got the minimum bias than others under logistic regression models. Conditioning on any confounding set through MSM had the same bias-reducing and produce unbiased estimations. In consideration of the standard error, we observed that adjusting for *A*_2_ by IPW-based-MSM resulted in higher precision in all situations. Thus, IPW-based-MSM produced unbiased causal effect estimations after conditioning on any *c-equivalent* set, and the strategy of adjusting for *A*_2_ achieved highest precision in this scenario. When using logistic regression models to adjust for confounders, adjusting for variables subset *A*_1_ produced the minimum bias.

## Discussion

In this paper, we focused on the 4 typical causal diagrams shown in Fig. 1 to assess the performances of logistic regression models and IPW-based-MSMs with respect to *c-equivalence*. The necessary and sufficient conditions for *T* and *Z* to be *c-equivalent* proposed by Pearl are that at least one of the following conditions hold [15]: 1) *T*_m_ = *Z*_m_; or 2) *T* and *Z* are G-admissible. Our results revealed that *c-equivalence* sets satisfying the *c-equivalenc*e condition 1) (e.g., *A*_2_ (*T*) and *A*_3_ (*Z, T*) in scenario 2) had different bias-reducing under logistic regression. For *c-equivalence* condition 2), adjusting for the set including all confounders had approximately bias-reducing as adjusting for the set containing the parent nodes of *Y*, while adjusting for the set containing the parent nodes of *X* was not equivalent to adjusting for the two above sets. However, under the framework of IPW-based-MSM, conditioning on any set of *c-equivalence*, as judged by the necessary and sufficient conditions, still had same bias-reducing. In summary, adjusting for different sets of *c-equivalence* under logistic regression always produced different bias-reducing; whereas when using IPW-based-MSM, the estimations of all strategies were approximately equivalent.

Adjusting more confounders would improve accuracy and precision of estimation in classic linear regression [28, 29]. Nevertheless, including more confounders in logistic regression model usually leads to less bias and lower precision [30]. Our studies showed that adjusting for the set containing the parent nodes of *X* had the minimum bias in logistic regression. With regard to the standard error, adjusting for set with fewer confounders would improve precision. Under the framework of IPW-based-MSM, we observed that adjusting for any set satisfying condition 2) had unbiased estimations; and conditioning on the set containing all parent nodes of *Y* achieved the highest precision in all situations. In summary, compared with logistic regression, the IPW-based-MSM produced unbiased causal effect estimates when the adjusted variable sets satisfied condition 2) and the optimal adjustment strategy was conditioning on parent nodes of outcome *Y*, which achieved the highest precision. Although the estimations obtained by logistic regression was biased, the estimation of adjusting for the parent nodes of the exposure *X* was nearest to true causal effect.

The true causal effect of exposure on outcome calculated by “*do-calculus*” is defined in terms of marginal probability distributions. However, the conditional treatment effects estimated from logistic regression model differ from the true causal effect [31, 32]. Logistic regression estimates do not behave like linear regression estimates. They are affected by omitted variables, even when those variables are unrelated to the independent variables in the model [11]. The use of IPW-based-MSM could lead to a more precise estimation of causal effects.

The discrepancy between the marginal OR and the conditional OR even in the absence of confounders is acknowledged as the non-collapsibility of the OR [4, 33]. The non-collapsibility effect depends on a variety of parameters, e.g., the effect of the exposure, the prevalence and effect of the covariate [4, 33]. According to our results, the differences in estimates between the logistic regression model and IPW-based-MSM were equal to the non-collapsibility effect in number. However, the discrepancy in estimates between these two model were different after adjusting for different sets of *c-equivalence* maybe due to these sets have different variables.

## Conclusions

In conclusion, the bias-reducing differed after adjusting for the sets of *c-equivalence* under the logistic regression model, whereas it were approximately equivalent when using IPW-based-MSM. All adjustment strategies through logistic regression were biased, while IPW-based-MSM could always obtain unbiased estimation when the adjusted set satisfied G-admissibility. Thus, for adjusting confounders set, we recommend IPW-based-MSM rather than logistic regression model.

## Additional files

Additional file 1: Appendix: Deducing whether *c-equivalence* had same bias-reducing potential under logistic regression model. (DOCX 107 kb)

Additional file 2: Figure S1. Scenario 1 (Fig. 1a), simulation results of the bias and standard error of *c-equivalence* sets *A*_1_ ≈ *A*_2_ ≈ *A*_3_ when varied across the log transformed odds ratio effect of *Z* on *X* and *X* on *Y*. (PDF 25 kb)

Additional file 3: Figure S2. Scenario 2 (Fig. 1b), simulation results of the bias and standard error of *c-equivalence* sets *A*_1_ ≈ *A*_2_ ≈ *A*_3_ when varied across the log transformed odds ratio effect of *Z* on *T* and *X* on *Y*. (PDF 25 kb)

Additional file 4: Figure S3. Scenario 2 (Fig. 1b), simulation results of the bias and standard error of *c-equivalence* sets *A*_1_ ≈ *A*_2_ ≈ *A*_3_ when varied across the log transformed odds ratio effect of *Z* on *X* and *W* on *X*. (PDF 25 kb)

Additional file 5: Figure S4. Scenario 2 (Fig. 1b), simulation results of the bias and standard error of *c-equivalenc*e sets *B*_1_ ≈ *B*_2_ when varied across the log transformed odds ratio effect of *Z* on *T* and *X* on *Y*. (PDF 19 kb)

Additional file 6: Figure S5. Scenario 2 (Fig. 1b), simulation results of the bias and standard error of *c-equivalence* sets *B*_1_ ≈ *B*_2_ when varied across the log transformed odds ratio effect of *Z* on *X* and *W* on *X*. (PDF 19 kb)

Additional file 7: Figure S6 Scenario 3 (Fig. 1c), simulation results of the bias and standard error of *c-equivalence* sets *A*_1_ ≈ *A*_2_ when varied across the log transformed odds ratio effect of *Z* on *X* and *W* on *V*. (PDF 19 kb)

Additional file 8: Figure S7. Scenario 3 (Fig. 1c), simulation results of the bias and standard error of *c-equivalence* sets *A*_1_ ≈ *A*_2_ when varied across the log transformed odds ratio effect of *T* on *Y*, *W* on *X* and *X* on *Y. (PDF 27 kb)*

Additional file 9: Figure S8. Scenario 3 (Fig. 1c), simulation results of the bias and standard error of *c-equivalence* sets *C*_1_ ≈ *C*_2_ ≈ *C*_3_ when varied across the log transformed odds ratio effect of *Z* on *X* and *W* on *V*. (PDF 25 kb)

Additional file 10: Figure S9. Scenario 3 (Fig. 1c), simulation results of the bias and standard error of *c-equivalence* sets *C*_1_ ≈ *C*_2_ ≈ *C*_3_ when varied across the log transformed odds ratio effect of *T* on *Y, W* on *X* and *X* on *Y*. (PDF 35 kb)

Additional file 11: Figure S10 Scenario 4 (Figure 1d), simulation results of the bias and standard error of *c-equivalence* sets *A*_1_ ≈ *A*_2_ ≈ *A*_3_ when varied across the log transformed odds ratio effect of *Z* on *X* and *X* on *Y*. (PDF 25 kb)

Additional file 12: Figure S11. Scenario 4 (Fig. 1d), simulation results of the bias and standard error of *c-equivalence* sets *A*_1_ ≈ *A*_2_ ≈ *A*_3_ when varied across the log transformed odds ratio effect of *Z* on *W*,*T* on *W* and *W* on *Y*. (PDF 35 kb)

## Abbreviations

*ACE*: Average causal effect
c-equivalence: Confounding equivalence
IPW-based-MSM: Inverse probability weight based marginal structural model
OR: Odds ratio
