# Supplementary material for: The alarming problems of confounding equivalence using logistic regression models in the perspective of causal diagrams
Source: BMC Med Res Methodol. 2017 Dec 28;17:177. doi: 10.1186/s12874-017-0449-7 (PMC5745640; doi:10.1186/s12874-017-0449-7)
Supplement: Supplementary file 1 — Appendix: Deducing whether c-equivalence had same bias-reducing potential under logistic regression model. (DOCX 107 kb) [file 12874_2017_449_MOESM1_ESM.docx]

**Appendix: Deducing whether *c-equivalence* had same bias-reducing potential under logistic regression model.**

Considered scenario 1 (Figure 1A) as a typical diagram. In this causal diagram, *A*_1_={*Z*}, *A*_2_={*T*} and *A*_3_={*Z, T*} composed the *c-equivalence* group that was sufficient to block all “back-door” paths from *X* to *Y* which was the second condition *c-equivalence*.

For *c-equivalence* sets *A*_1_*≈A*_2_*≈A*_3_, the average causal effect ($ACE^{\log(OR)}$) of *X* on *Y* was calculated as $ACE^{\log(OR)}=\text{logit} E[Y|do(X=1)]-\text{logit} E[Y|do(X=0)]$,

where$E[Y|do(X=1)]=P[Y=1|do(X=1)]=\sum_{T,Z} P(Y=1|X=1,T)P(T|Z)P(Z)$

$E[Y|do(X=0)]=P[Y=1|do(X=0)]=\sum_{T,Z} P(Y=1|X=0,T)P(T|Z)P(Z)$.

Then,$ACE^{\log(OR)}=\ln\left[\frac{\sum_{T,Z} P(Y=1|X=1,T)P(T|Z)P(Z)\sum_{T,Z} P(Y=0|X=0,T)P(T|Z)P(Z)}{\sum_{T,Z} P(Y=0|X=1,T)P(T|Z)P(Z)\sum_{T,Z} P(Y=1|X=0,T)P(T|Z)P(Z)}\right]$

Let$COR=\frac{\sum_{T,Z} P(Y=1|X=1,T)P(T|Z)P(Z)\sum_{T,Z} P(Y=0|X=0,T)P(T|Z)P(Z)}{\sum_{T,Z} P(Y=0|X=1,T)P(T|Z)P(Z)\sum_{T,Z} P(Y=1|X=0,T)P(T|Z)P(Z)}$,

which could represent the causal effect on the scale of odds ratio (OR).

By conditioning on *A*_1_={*Z*}, the effect of *X* on *Y* was equal to

$$\begin{matrix} \beta_{X}^{A_{1}}=\log it P(Y=1|X=1,Z)-\log it P(Y=1|X=0,Z) \\ \text{=ln}\left[\frac{\sum_{T} P(Y=1|X=1,T)P(T|Z)\sum_{T} P(Y=0|X=0,T)P(T|Z)}{\sum_{T} P(Y=0|X=1,T)P(T|Z)\sum_{T} P(Y=1|X=0,T)P(T|Z)}\right] \end{matrix}$$

Let$OR_{Z}=\frac{\sum_{T} P(Y=1|X=1,T)P(T|Z)\sum_{T} P(Y=0|X=0,T)P(T|Z)}{\sum_{T} P(Y=0|X=1,T)P(T|Z)\sum_{T} P(Y=1|X=0,T)P(T|Z)}$,

which represented the effect after adjusting for *A*_1_ on the scale of the OR. Then, we could calculate that$OR_{Z}=\frac{\exp(c_{3})C}{D}$, where

$C=k_{11}+\exp(c_{2})k_{12}+k_{21}+k_{22}$; $D=k_{11}+k_{12}+\exp(c_{2})k_{21}+k_{22}$

$$k_{11}=\frac{\exp(c_{0}Z+\alpha_{1})}{1+\exp(c_{0}Z+\alpha_{1})}\frac{\exp(c_{0}Z+\alpha_{1})}{1+\exp(c_{0}Z+\alpha_{1})}\frac{\exp(c_{2}+\alpha_{0})}{1+\exp(c_{2}+\alpha_{0})}\frac{1}{1+\exp(c_{2}+c_{3}+\alpha_{0})}$$

$$k_{12}=\frac{1}{1+\exp(c_{2}+c_{3}+\alpha_{0})}\frac{\exp(c_{0}Z+\alpha_{1})}{1+\exp(c_{0}Z+\alpha_{1})}\frac{\exp(\alpha_{0})}{1+\exp(\alpha_{0})}\frac{1}{1+\exp(c_{0}Z+\alpha_{1})}$$

$$k_{21}=\frac{1}{1+\exp(c_{3}+\alpha_{0})}\frac{1}{1+\exp(c_{0}Z+\alpha_{1})}\frac{\exp(\alpha_{0})}{1+\exp(c_{2}+\alpha_{0})}\frac{\exp(c_{0}Z+\alpha_{1})}{1+\exp(c_{0}Z+\alpha_{1})}$$

$$k_{22}=\frac{1}{1+\exp(c_{3}+\alpha_{0})}\frac{1}{1+\exp(c_{0}Z+\alpha_{1})}\frac{\exp(\alpha_{0})}{1+\exp(\alpha_{0})}\frac{1}{1+\exp(c_{0}Z+\alpha_{1})}$$

Similarly, the effect of *X* on *Y*, when conditioning on *A*_2_*=*{*T*}, was equal to

$\begin{matrix} \beta_{X}^{A_{2}}=\log it P(Y=1|X=1,T)-\log it P(Y=1|X=0,T) \\ \text{=ln}\left[\frac{P(Y=1|X=1,T)P(Y=0|X=0,T)}{P(Y=0|X=1,T)P(Y=1|X=0,T)}\right] \\ =c_{3} \end{matrix}$.

Furthermore, the effect of *X* on *Y*, when conditioning on *A*_3_={*T*, *Z*}, was

$\begin{matrix} \beta_{X}^{A_{3}}=\log it P(Y=1|X=1,T,Z)-\log it P(Y=1|X=0,T,Z) \\ =\ln\left[\frac{P(Y=1|X=1,T)P(Y=0|X=0,T)}{P(Y=0|X=1,T)P(Y=1|X=0,T)}\right] \\ =c_{3} \end{matrix}$.

To determine whether $\beta_{X}^{A_{1}}-\beta=\beta_{X}^{A_{2}}-\beta=\beta_{X}^{A_{3}}-\beta$ for *A*_1_*≈A*_2_*≈A*_3_, we need to compare $\beta_{X}^{A_{1}}$, $\beta_{X}^{A_{2}}$and $\beta_{X}^{A_{3}}$. Easily, we easily obtained$\beta_{X}^{A_{2}}\text{=}\beta_{X}^{A_{3}}\text{=}c_{3}$.

Then, we compared $\beta_{X}^{A_{1}}$with$c_{3}$,

$$\beta_{X}^{A_{1}}-c_{3}=\ln[OR_{Z}]-\ln[\exp(c_{3})]=\ln[\frac{OR_{Z}}{\exp(c_{3})}]\text{=}\ln\left[\frac{k_{11}+\exp(c_{2})k_{12}+k_{21}+k_{22}}{k_{11}+k_{12}+\exp(c_{2})k_{21}+k_{22}}\right]$$

and compared $\frac{k_{11}+\exp(c_{2})k_{12}+k_{21}+k_{22}}{k_{11}+k_{12}+\exp(c_{2})k_{21}+k_{22}}$ with 1.

Because $k_{11}+\exp(c_{2})k_{12}+k_{21}+k_{22}>0$ and $k_{11}+k_{12}+\exp(c_{2})k_{21}+k_{22}>0$, we only need to compare $\exp(c_{2})k_{12}+k_{21}$ with $k_{12}+\exp(c_{2})k_{21}$

If $c_{2}=0$ or $c_{3}=0$, $\beta_{X}^{A_{1}}\text{=}c_{3}$

If $c_{2}\neq0$and $c_{3}>0$, $\beta_{X}^{A_{1}}\text{<}c_{3}$

If $c_{2}\neq0$ and $c_{3}<0$, $\beta_{X}^{A_{1}}>c_{3}$

According the deduction above, we could see that

If $c_{2}=0$ or $c_{3}=0$,$\beta_{X}^{A_{1}}\text{=}\beta_{X}^{A_{2}}\text{=}\beta_{X}^{A_{3}}\text{=}c_{3}$

If $c_{2}\neq0$and $c_{3}>0$,$\beta_{X}^{A_{1}}<\beta_{X}^{A_{2}}\text{=}\beta_{X}^{A_{3}}\text{=}c_{3}$

If $c_{2}\neq0$ and $c_{3}<0$,$\beta_{X}^{A_{1}}>\beta_{X}^{A_{2}}\text{=}\beta_{X}^{A_{3}}\text{=}c_{3}$

We obtained $\beta_{X}^{A_{2}}\text{=}\beta_{X}^{A_{3}}$ under all conditions, suggesting that the bias-reducing of *c-equivalence* *A_2_*≈*A*_3_ was always equivalent under the logistic regression model. By contrast, $\beta_{X}^{A_{1}}\text{=}\beta_{X}^{A_{2}}\text{=}\beta_{X}^{A_{3}}$ only if $c_{2}=0$ or $c_{3}=0$, indicating that the bias-reducing of *c-equivalence* *A*_1_≈*A_2_*≈*A*_3_ remained equivalent under the logistic regression model in this situation. However, $\beta_{X}^{A_{1}}\text{<}\beta_{X}^{A_{2}}\text{=}\beta_{X}^{A_{3}}$ if $c_{2}\neq0$and $c_{3}>0$; and$\beta_{X}^{A_{1}}\text{>}\beta_{X}^{A_{2}}\text{=}\beta_{X}^{A_{3}}$ if $c_{2}\neq0$ and $c_{3}<0$ indicating that the unequal bias-reducing of the *c-equivalence* of *A*_1_≈*A_2_*≈*A*_3_ no longer held when both$c_{2}$and$c_{3}$were not equal to zero.
