# Supplementary figures and images for: The alarming problems of confounding equivalence using logistic regression models in the perspective of causal diagrams
Source: BMC Med Res Methodol. 2017 Dec 28;17:177. doi: 10.1186/s12874-017-0449-7 (PMC5745640; doi:10.1186/s12874-017-0449-7)

(A)

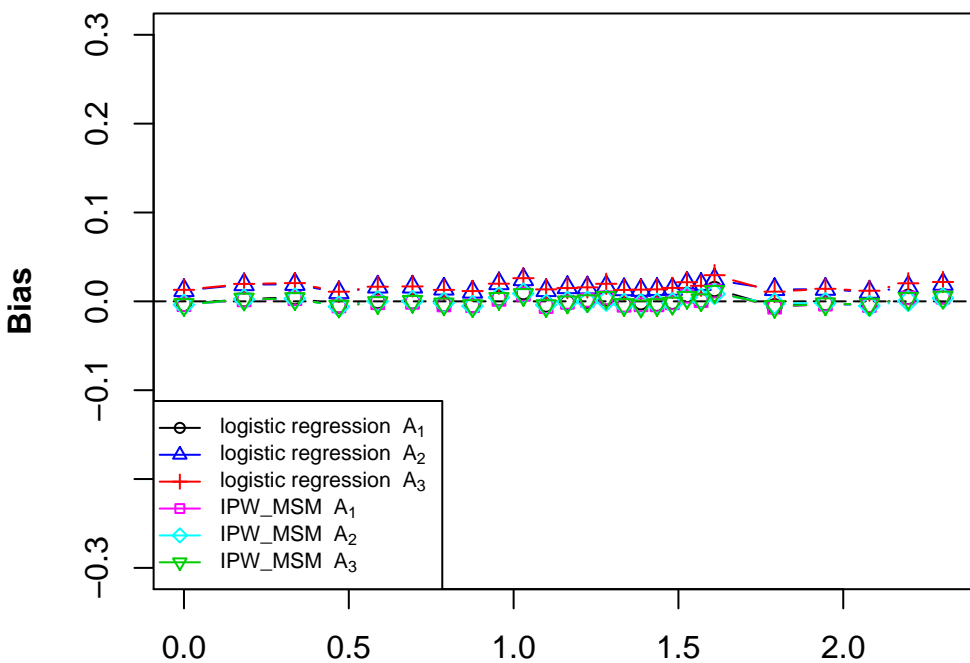

(B)

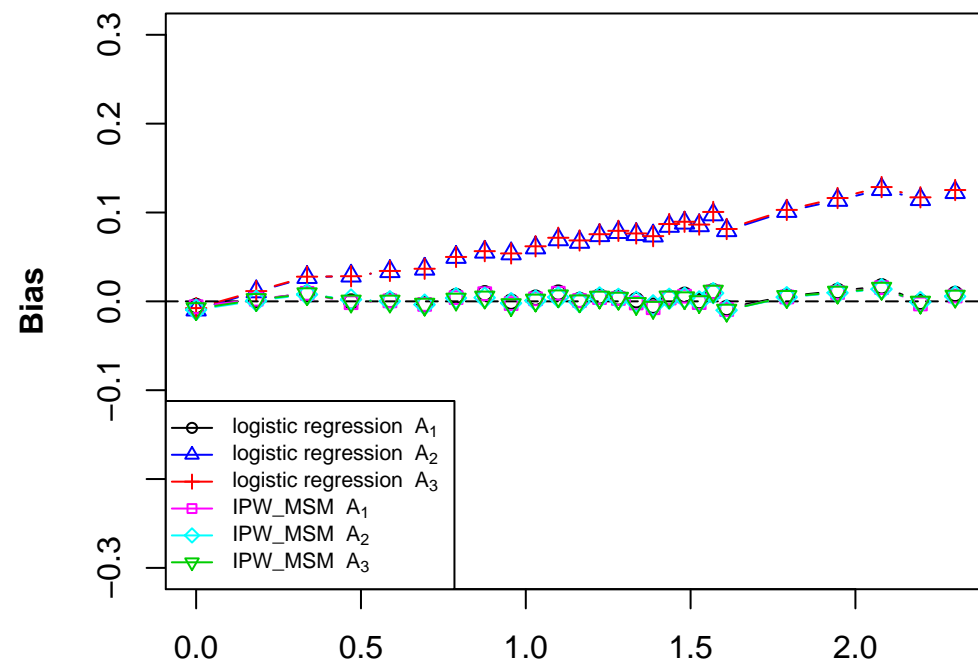

(C)

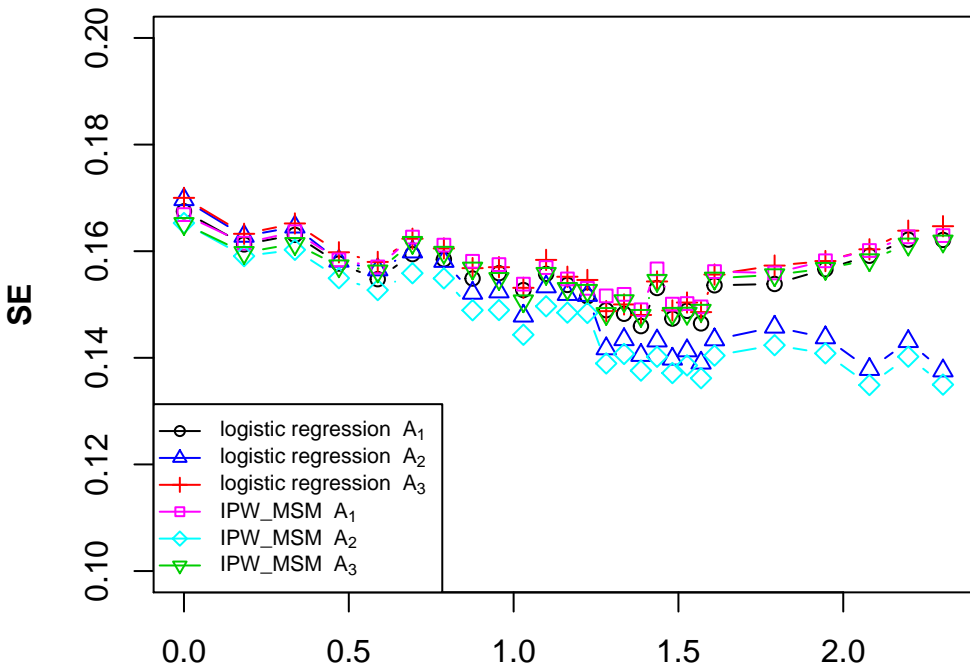

(D)

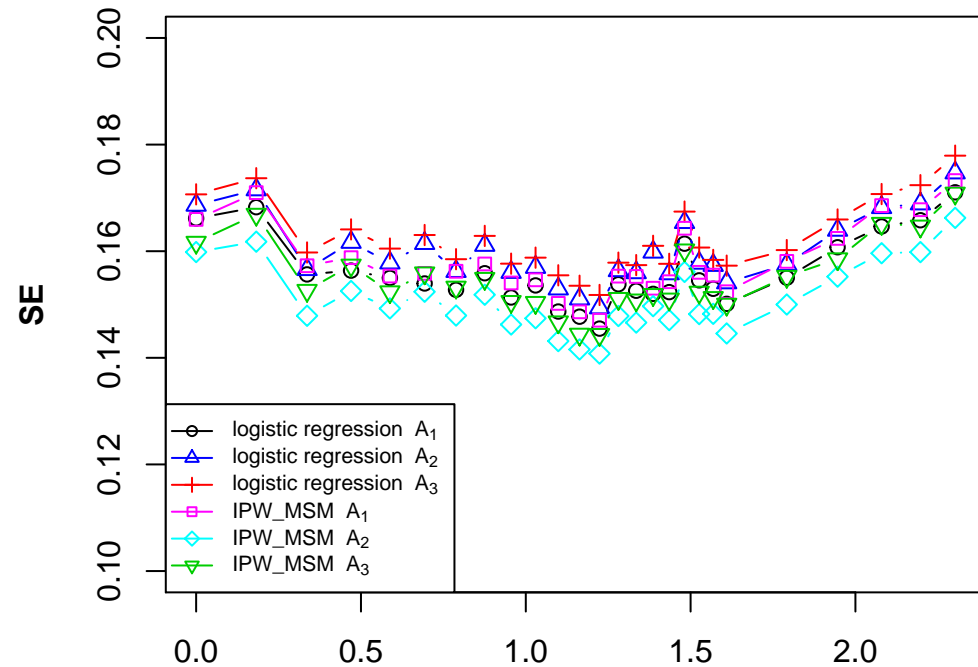

Supplement: Supplementary file 2 — Scenario 1 (Fig. 1a), simulation results of the bias and standard error of c-equivalence sets A 1 ≈ A 2 ≈ A 3 when varied across the log transformed odds ratio effect of Z on X and X on Y. (PDF 25 kb) [file 12874_2017_449_MOESM2_ESM.pdf]

(A)

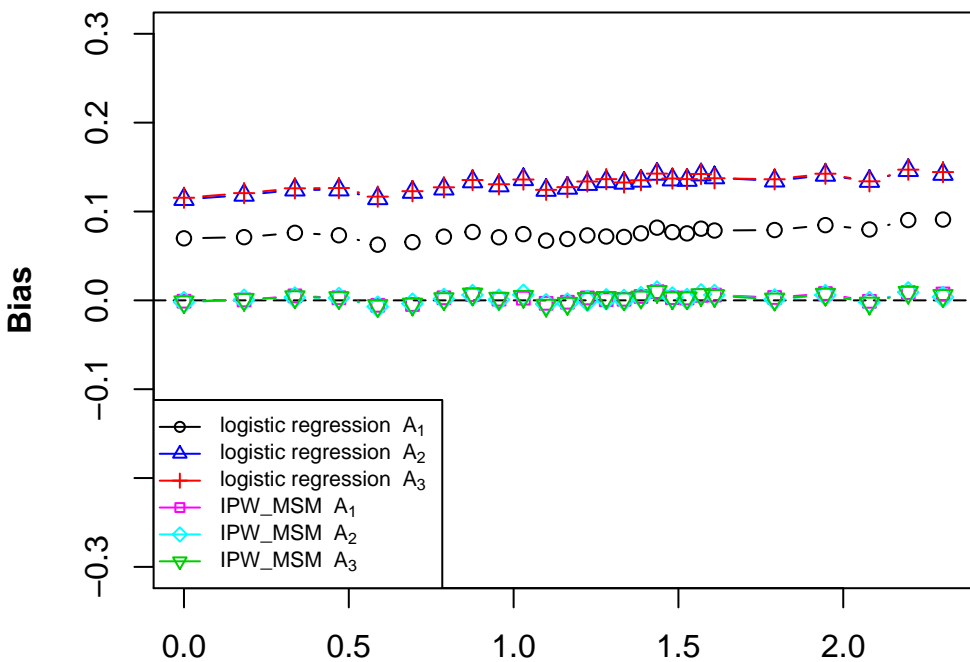

(B)

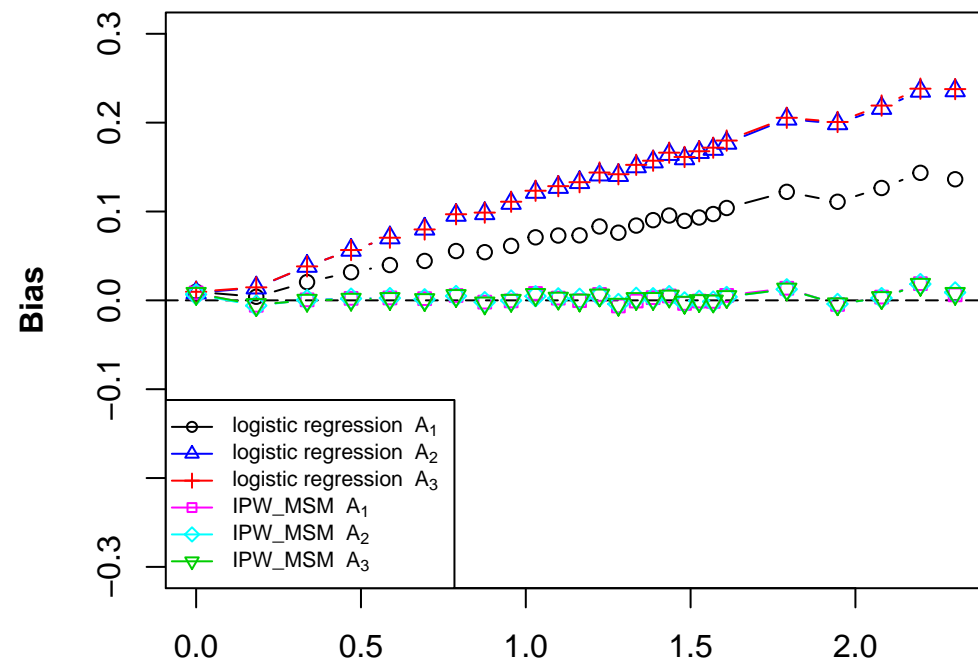

(C)

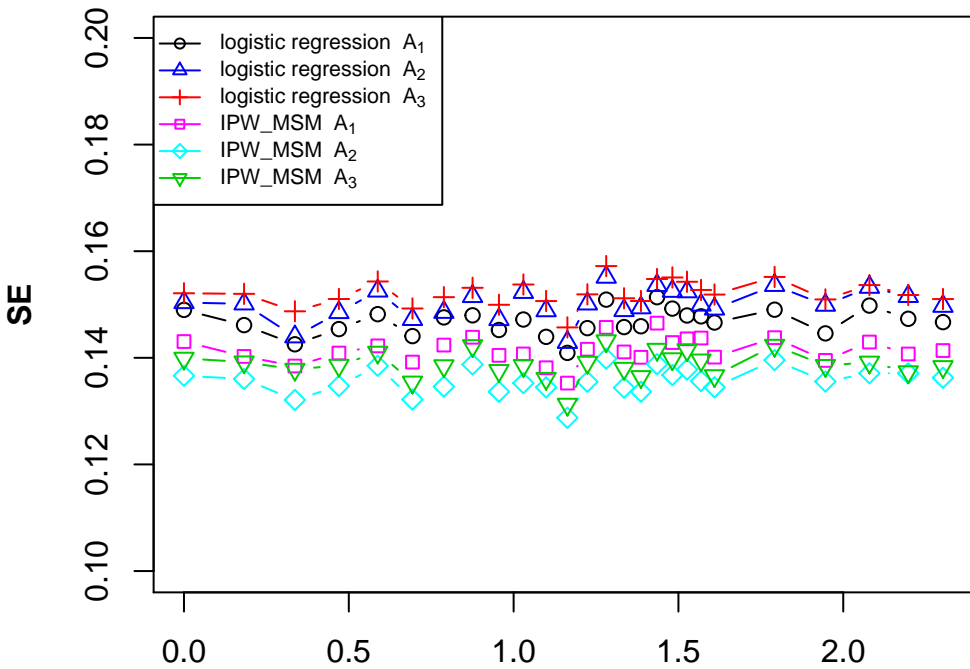

(D)

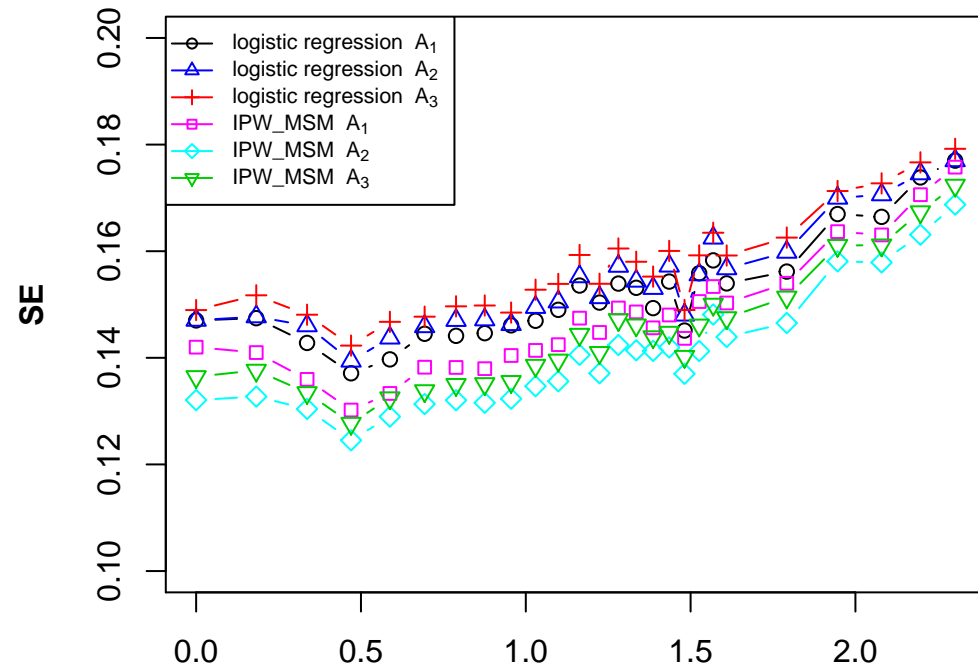

Supplement: Supplementary file 3 — Scenario 2 (Fig. 1b), simulation results of the bias and standard error of c-equivalence sets A 1 ≈ A 2 ≈ A 3 when varied across the log transformed odds ratio effect of Z on T and X on Y. (PDF 25 kb) [file 12874_2017_449_MOESM3_ESM.pdf]

(A)

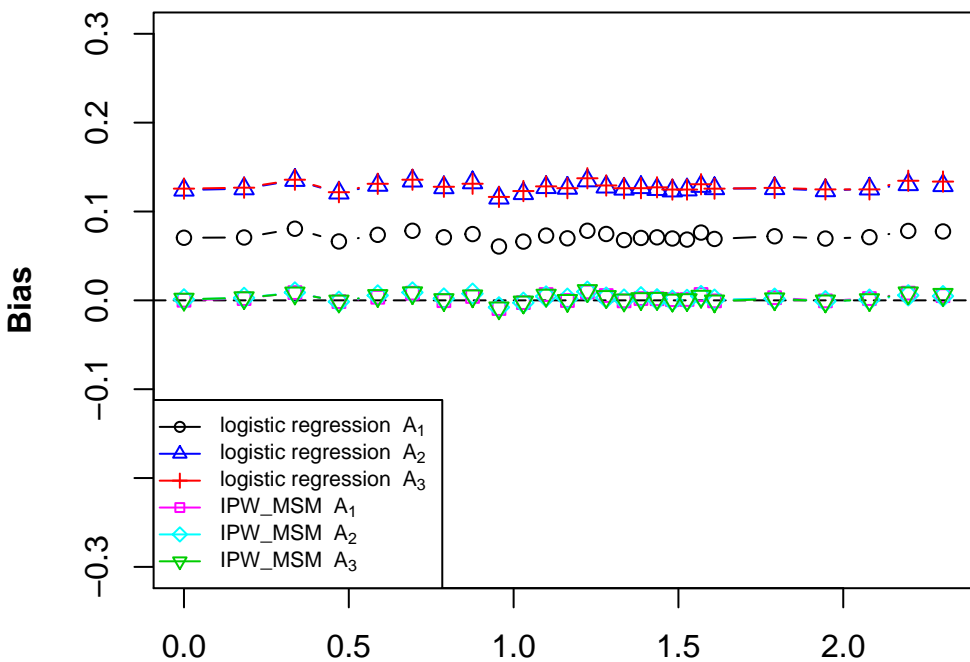

(B)

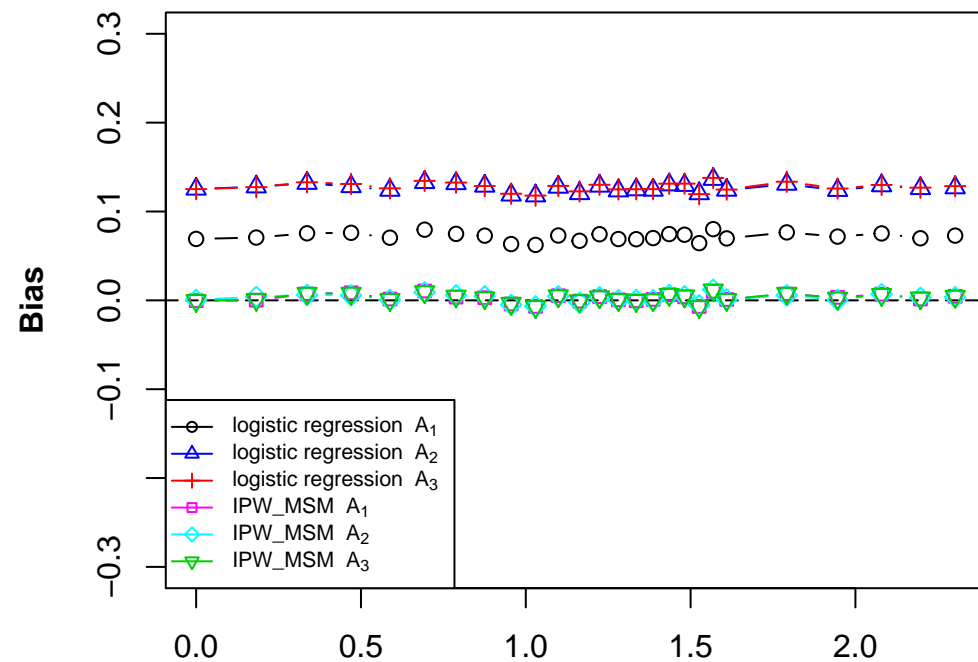

(C)

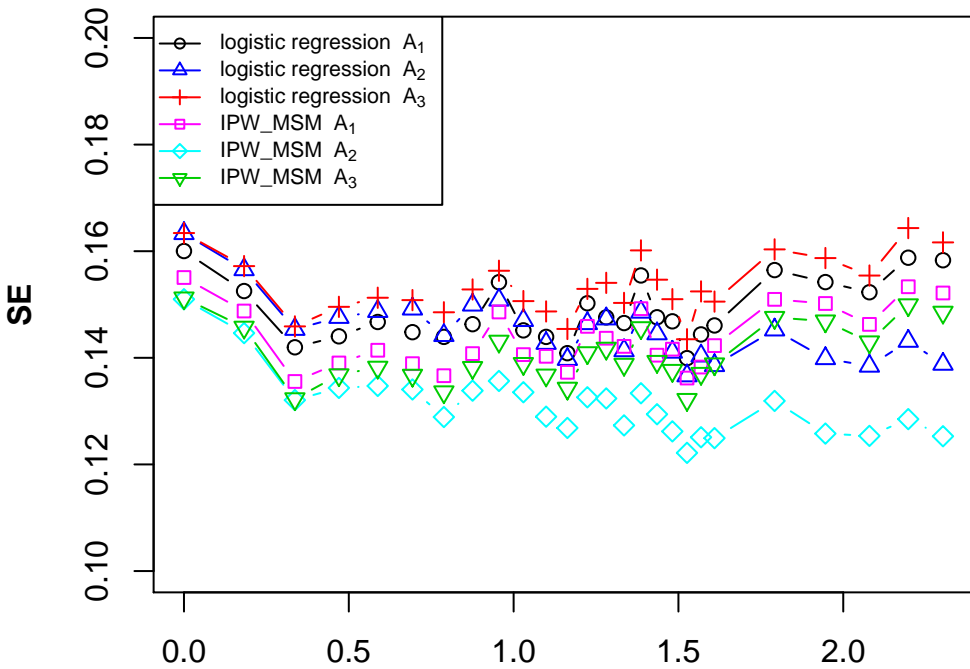

(D)

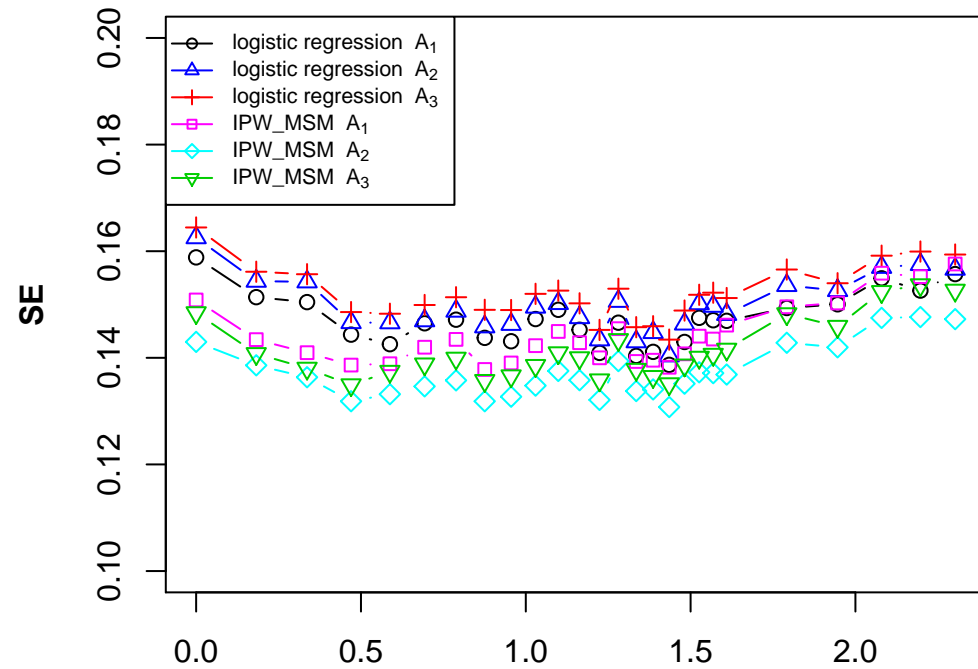

Supplement: Supplementary file 4 — Scenario 2 (Fig. 1b), simulation results of the bias and standard error of c-equivalence sets A 1 ≈ A 2 ≈ A 3 when varied across the log transformed odds ratio effect of Z on X and W on X. (PDF 25 kb) [file 12874_2017_449_MOESM4_ESM.pdf]

(A)

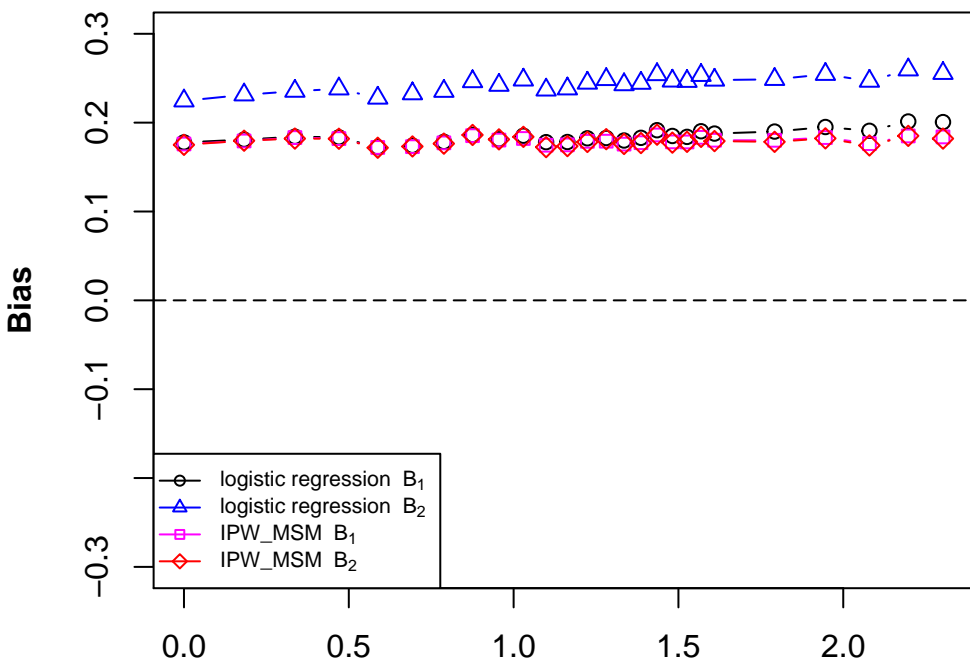

(B)

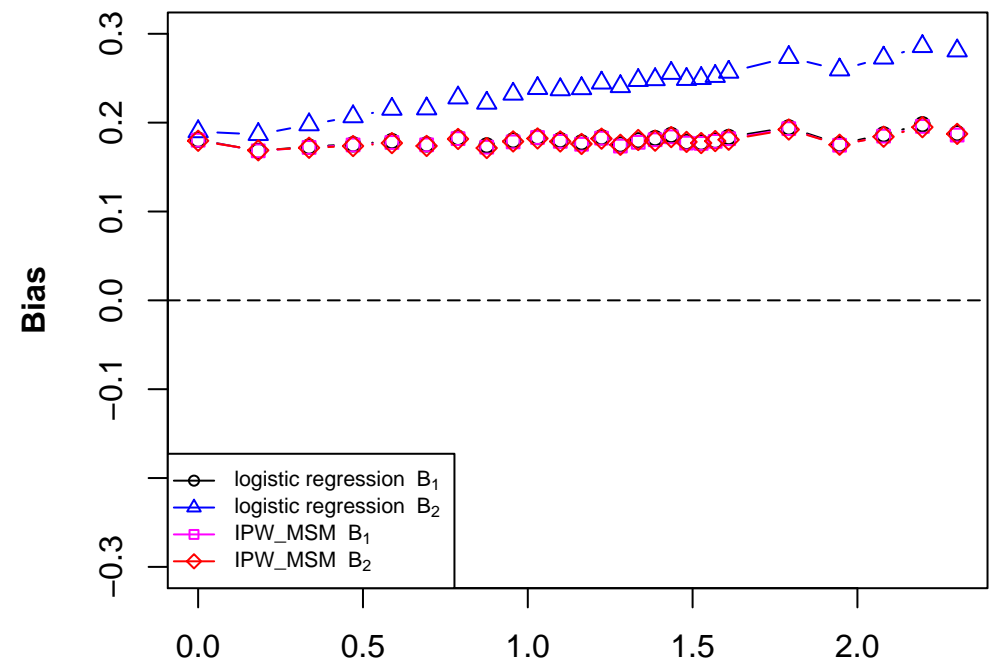

(C)

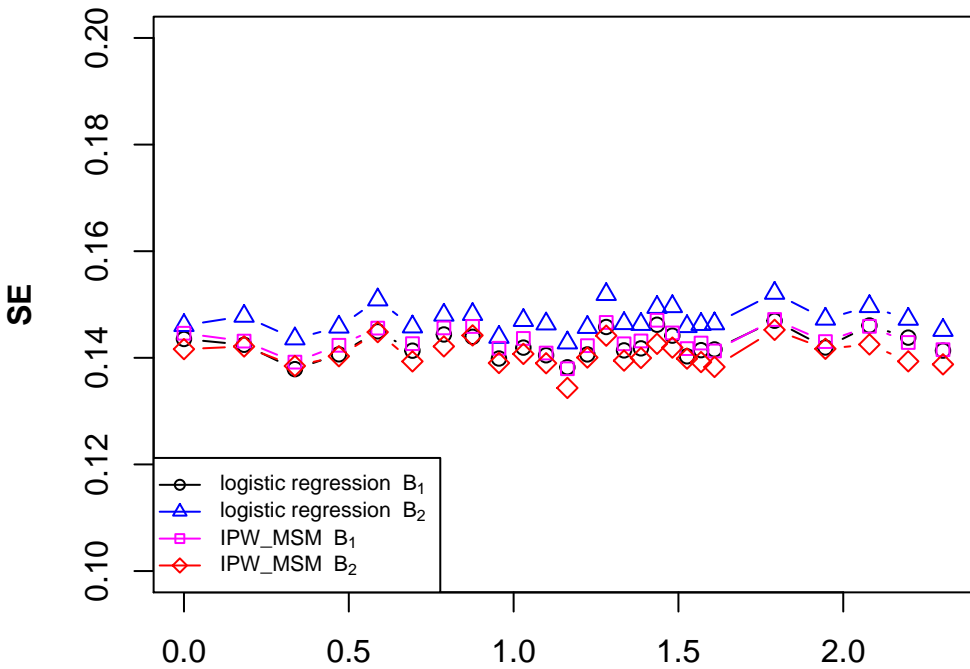

(D)

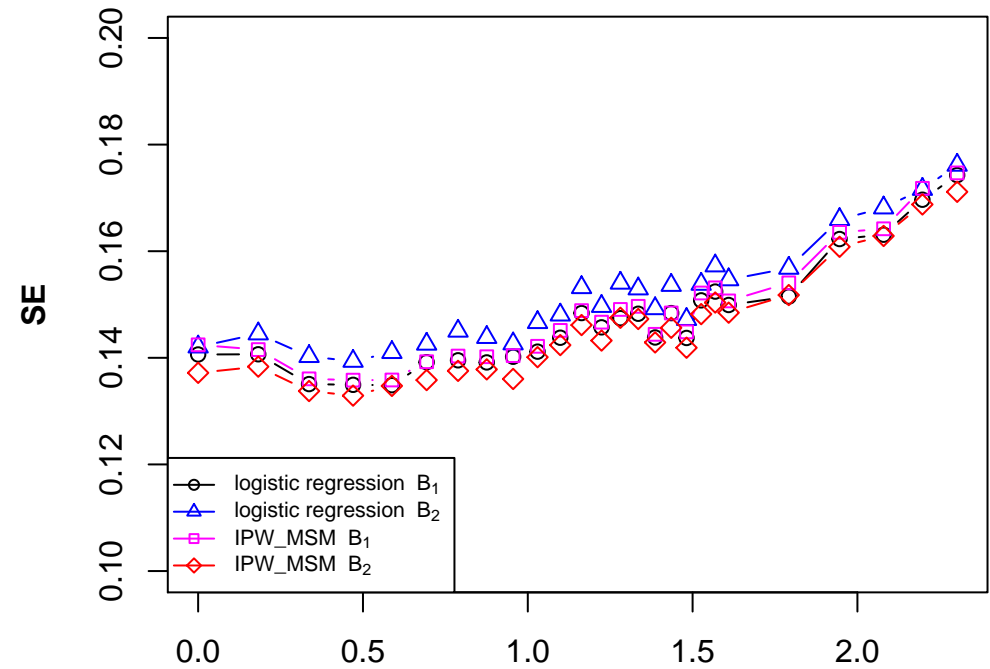

Supplement: Supplementary file 5 — Scenario 2 (Fig. 1b), simulation results of the bias and standard error of c-equivalence sets B 1 ≈ B 2 when varied across the log transformed odds ratio effect of Z on T and X on Y. (PDF 19 kb) [file 12874_2017_449_MOESM5_ESM.pdf]

(A)

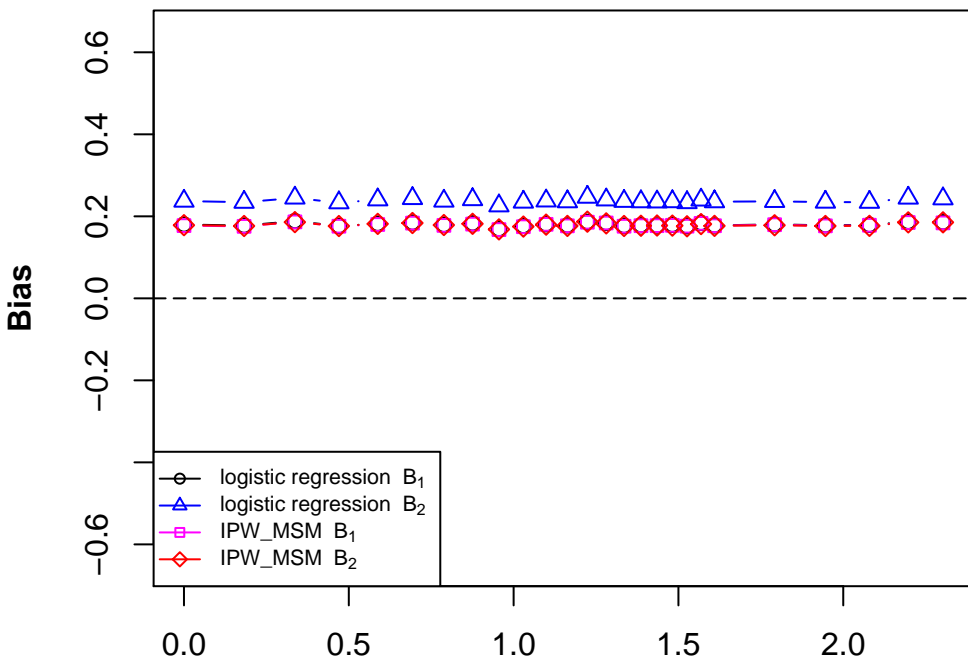

(B)

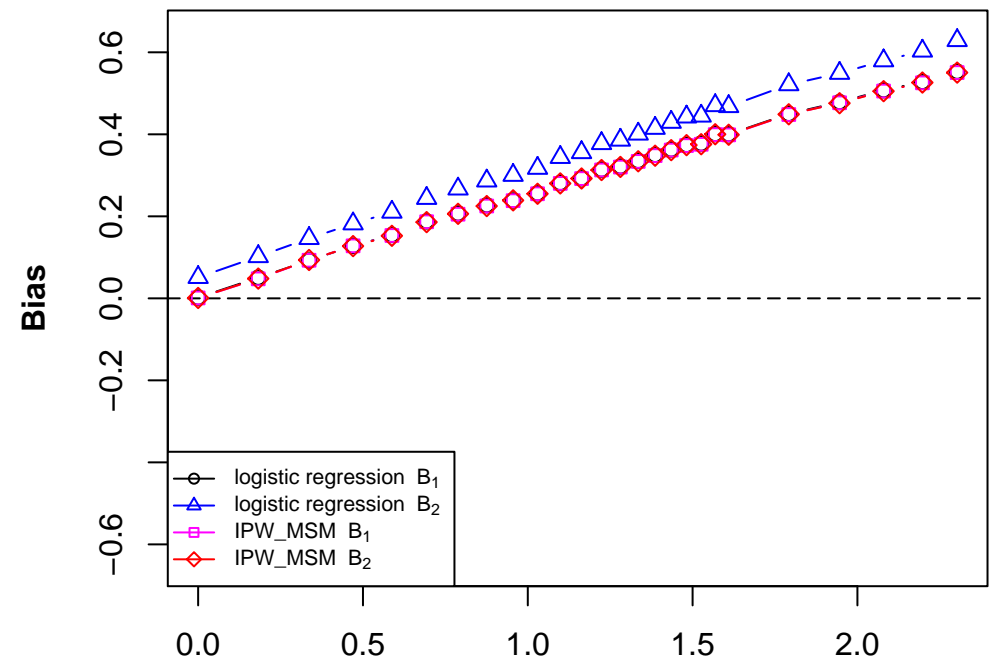

(C)

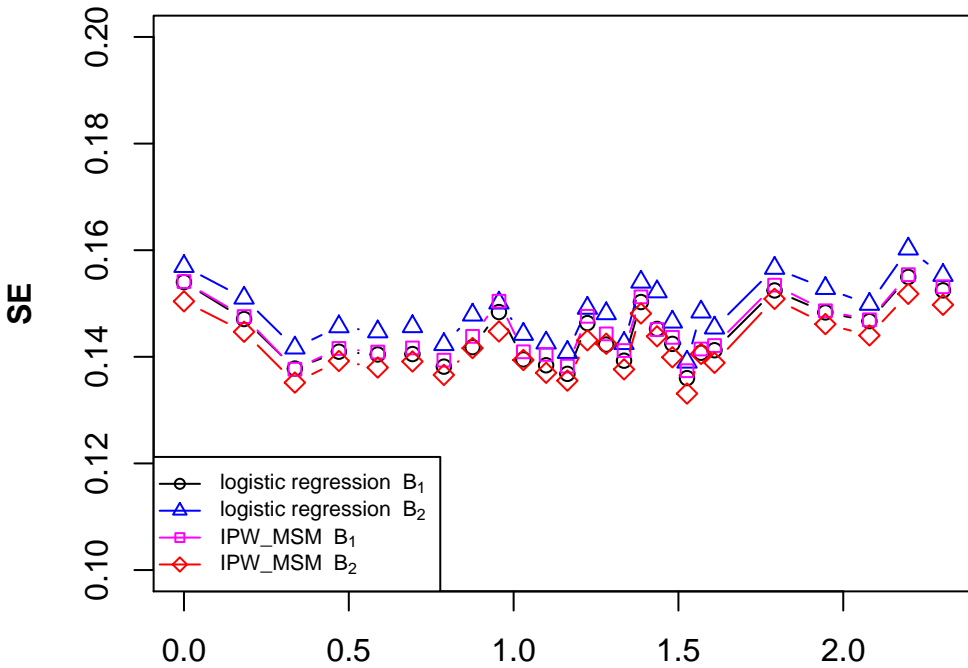

(D)

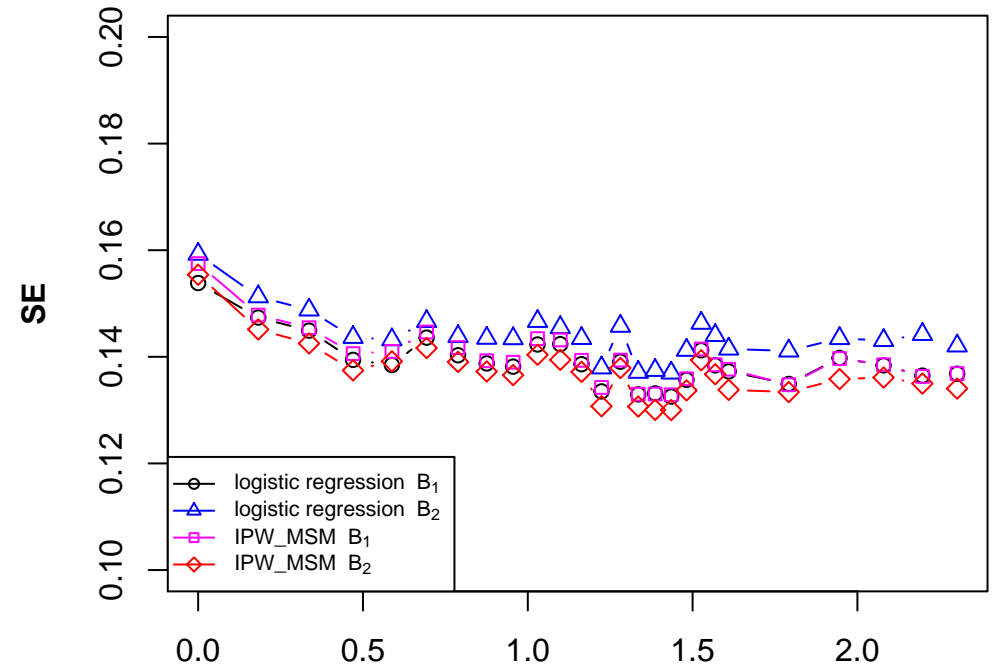

Supplement: Supplementary file 6 — Scenario 2 (Fig. 1b), simulation results of the bias and standard error of c-equivalence sets B 1 ≈ B 2 when varied across the log transformed odds ratio effect of Z on X and W on X. (PDF 19 kb) [file 12874_2017_449_MOESM6_ESM.pdf]

(A)

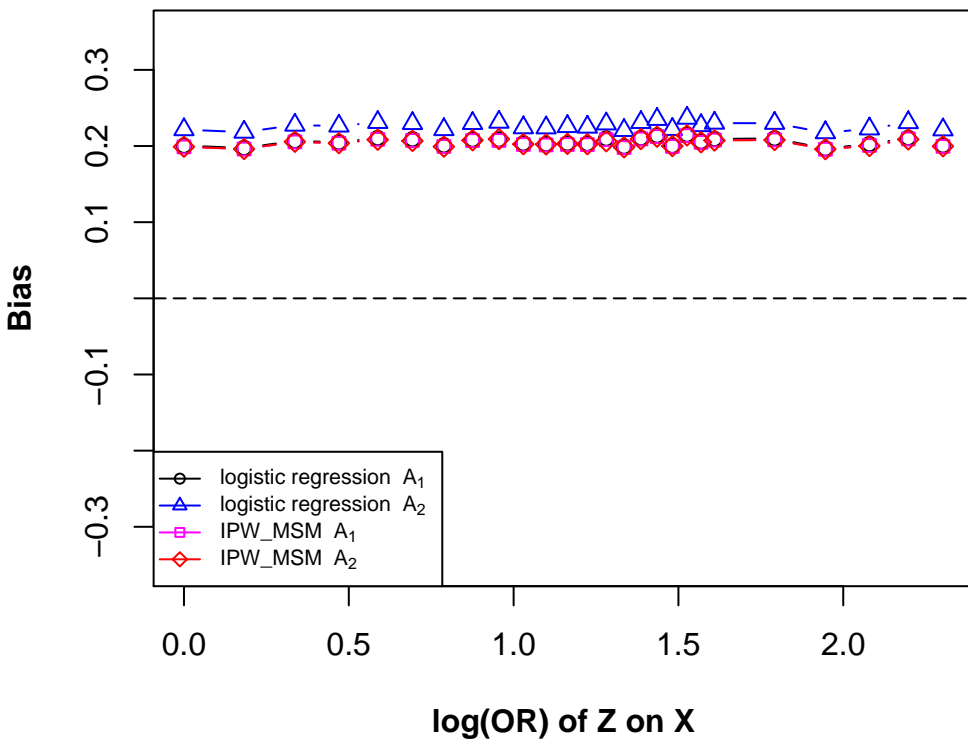

(B)

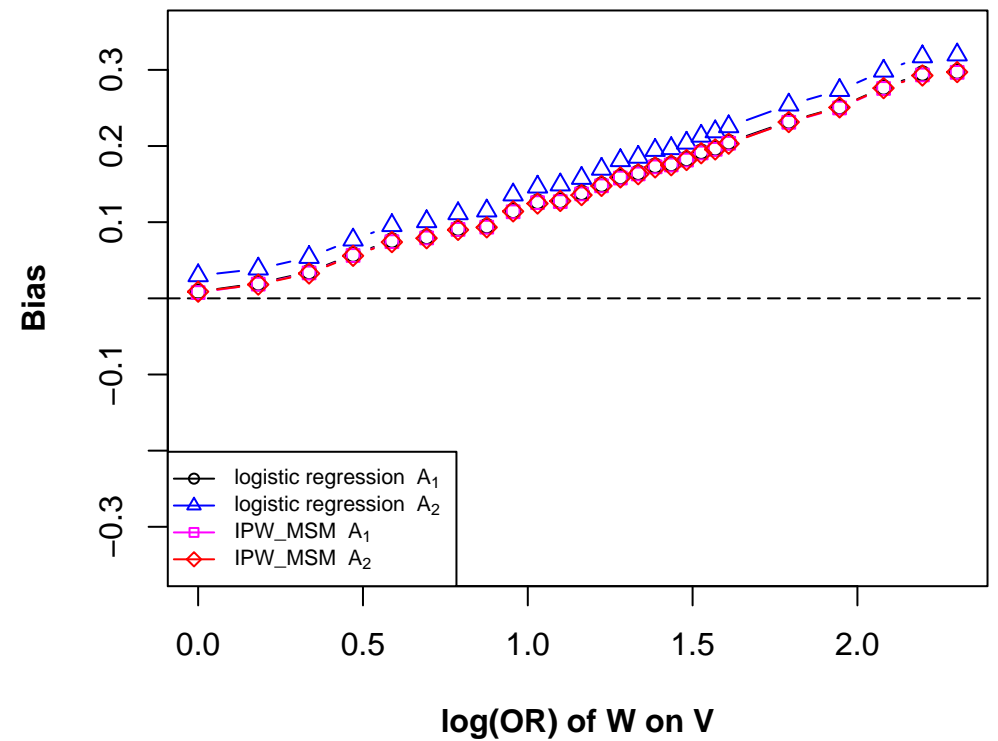

(C)

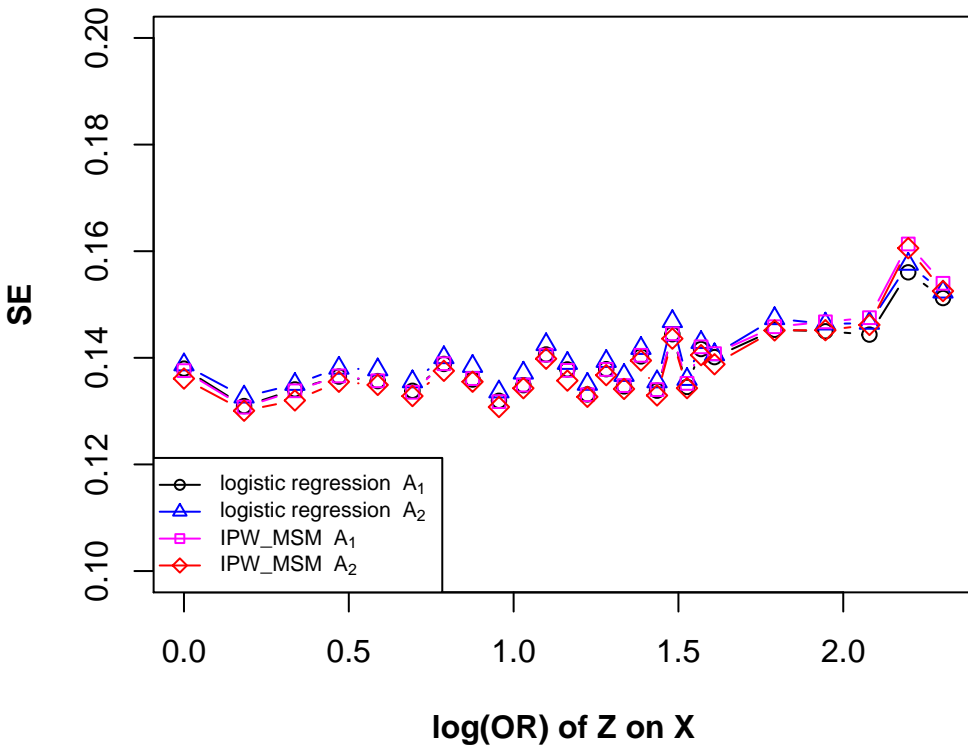

(D)

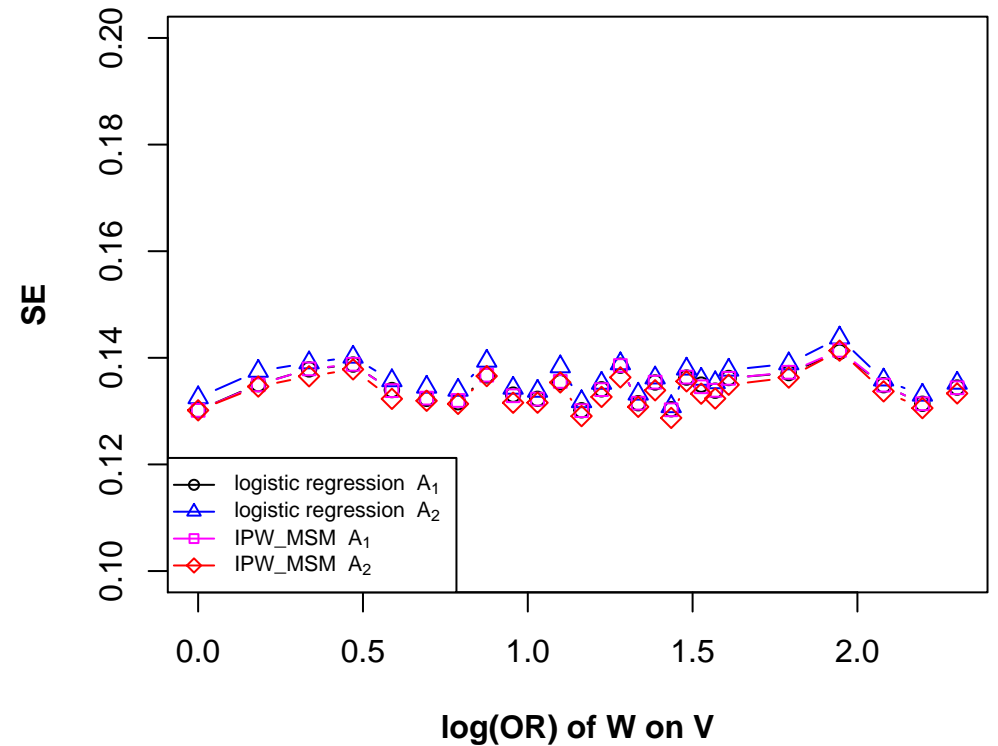

Supplement: Supplementary file 7 — Scenario 3 (Fig. 1c), simulation results of the bias and standard error of c-equivalence sets A 1 ≈ A 2 when varied across the log transformed odds ratio effect of Z on X and W on V. (PDF 19 kb) [file 12874_2017_449_MOESM7_ESM.pdf]

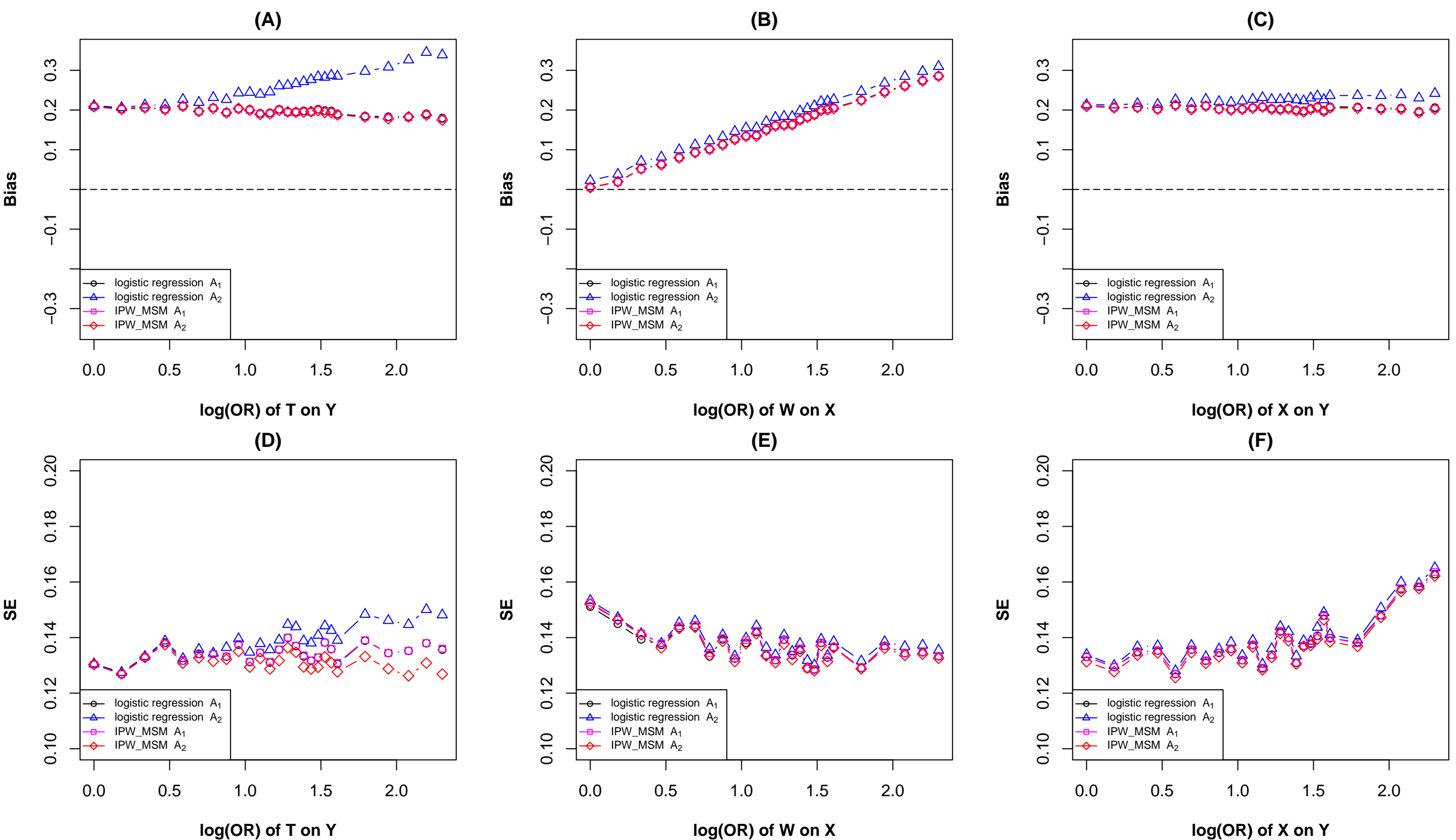

Supplement: Supplementary file 8 — Scenario 3 (Fig. 1c), simulation results of the bias and standard error of c-equivalence sets A 1 ≈ A 2 when varied across the log transformed odds ratio effect of T on Y, W on X and X on Y. (PDF 27 kb) [file 12874_2017_449_MOESM8_ESM.pdf]

(A)

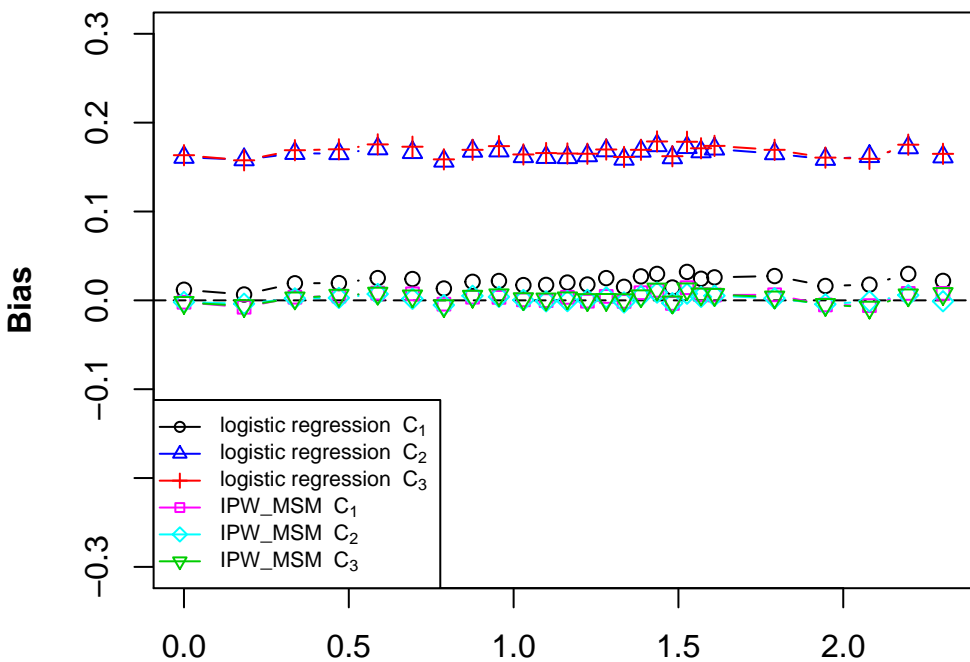

(B)

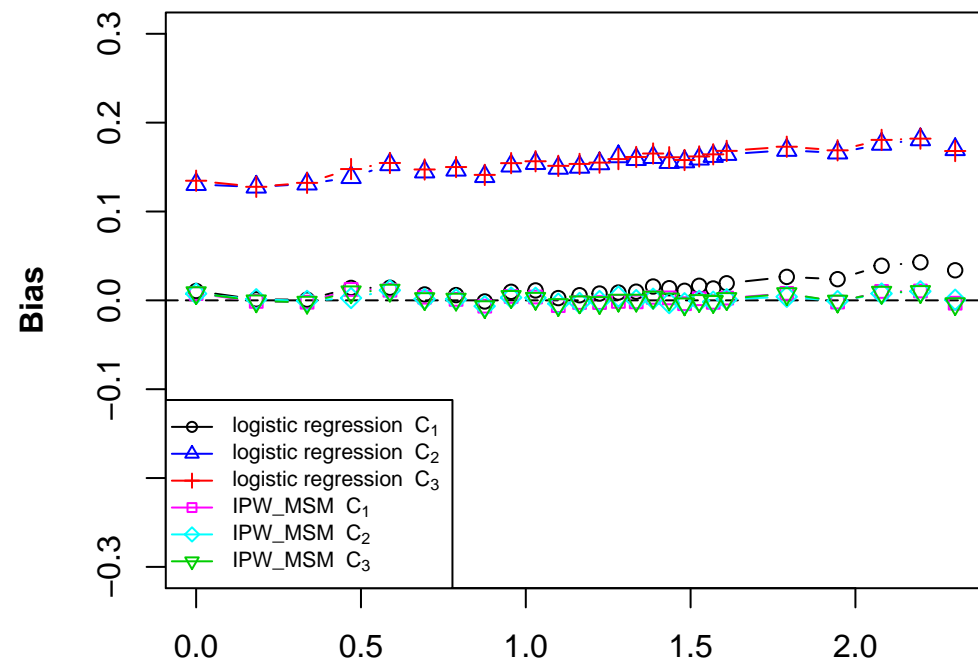

(C)

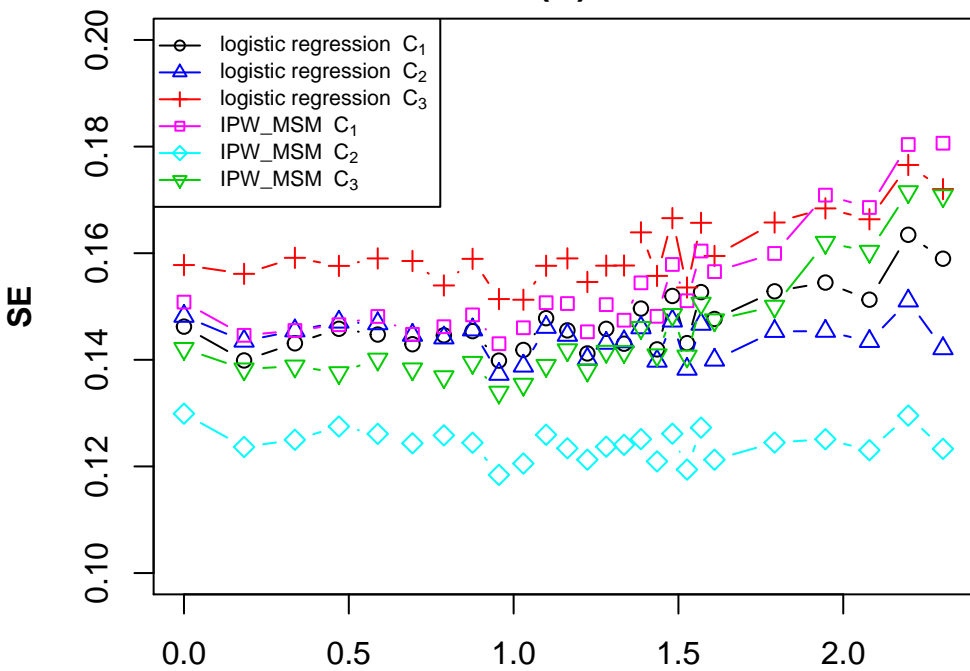

(D)

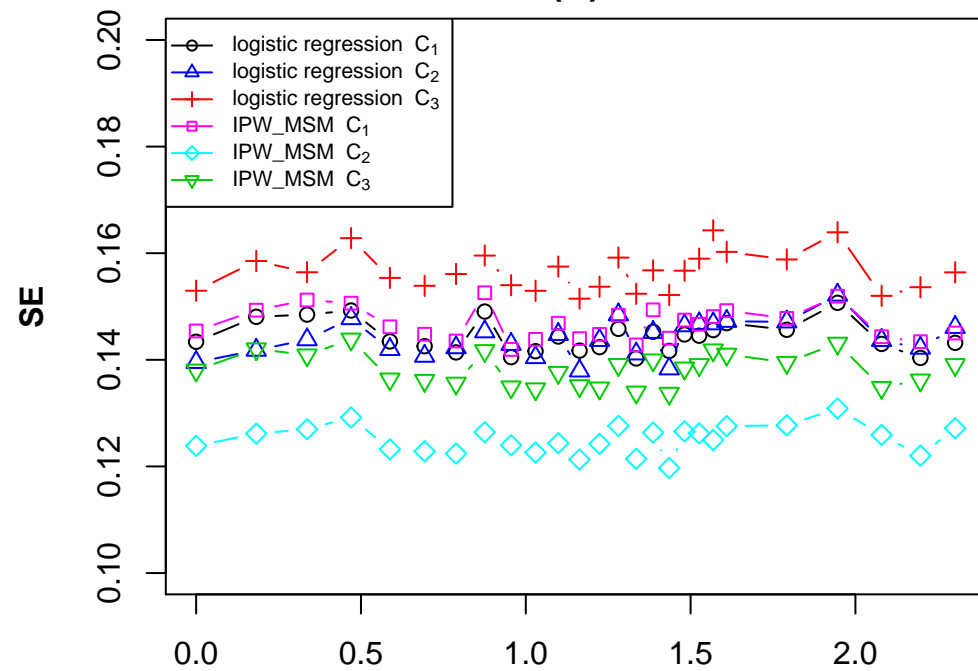

Supplement: Supplementary file 9 — Scenario 3 (Fig. 1c), simulation results of the bias and standard error of c-equivalence sets C 1 ≈ C 2 ≈ C 3 when varied across the log transformed odds ratio effect of Z on X and W on V. (PDF 25 kb) [file 12874_2017_449_MOESM9_ESM.pdf]

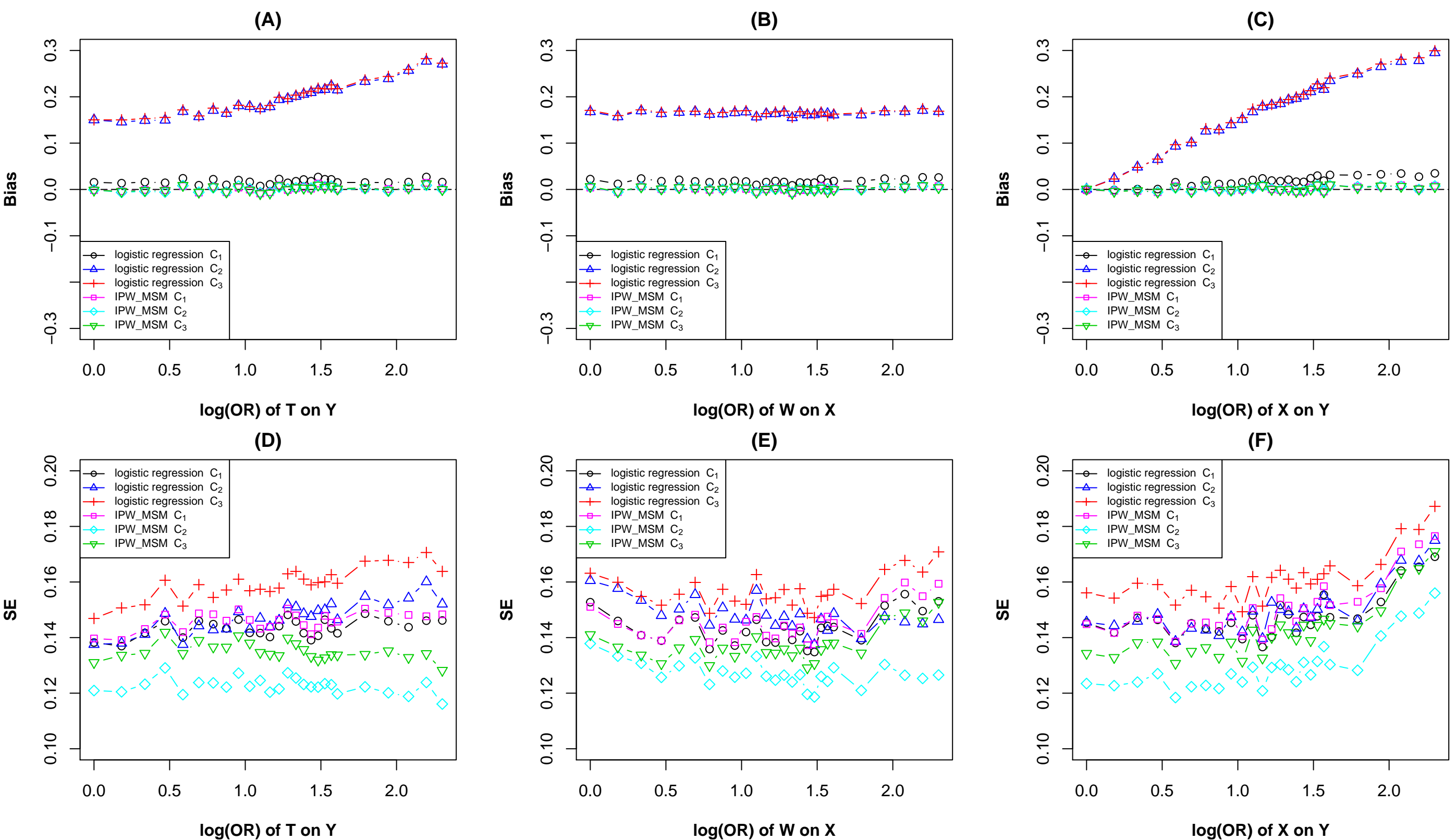

Supplement: Supplementary file 10 — Scenario 3 (Fig. 1c), simulation results of the bias and standard error of c-equivalence sets C 1 ≈ C 2 ≈ C 3 when varied across the log transformed odds ratio effect of T on Y, W on X and X on Y. (PDF 35 kb) [file 12874_2017_449_MOESM10_ESM.pdf]

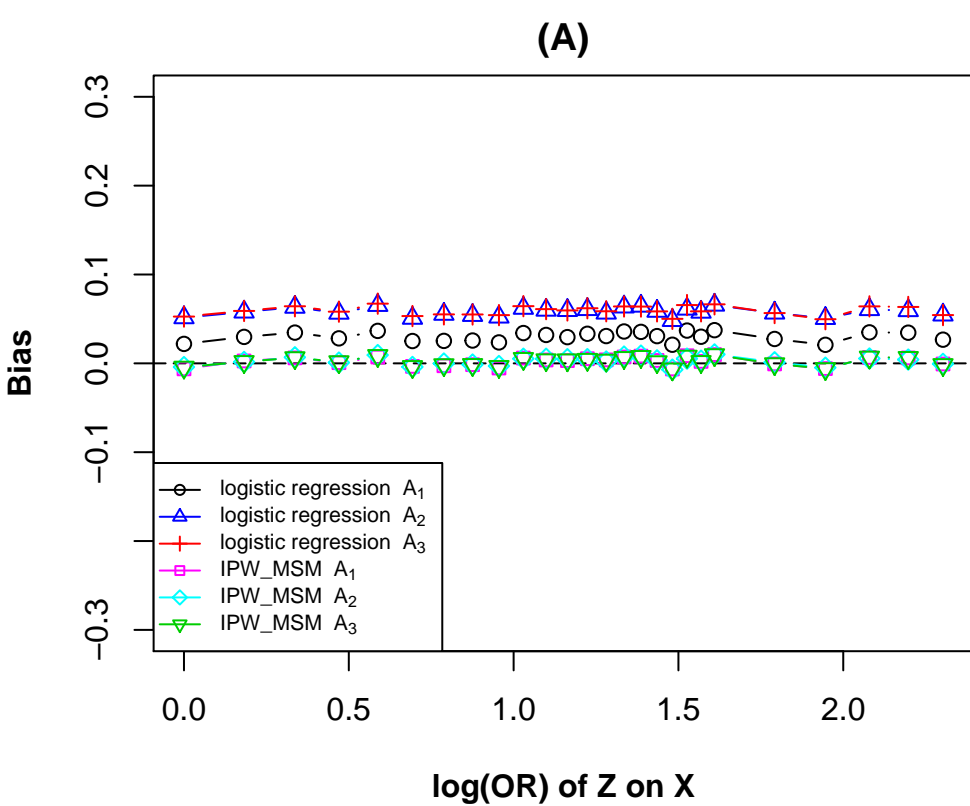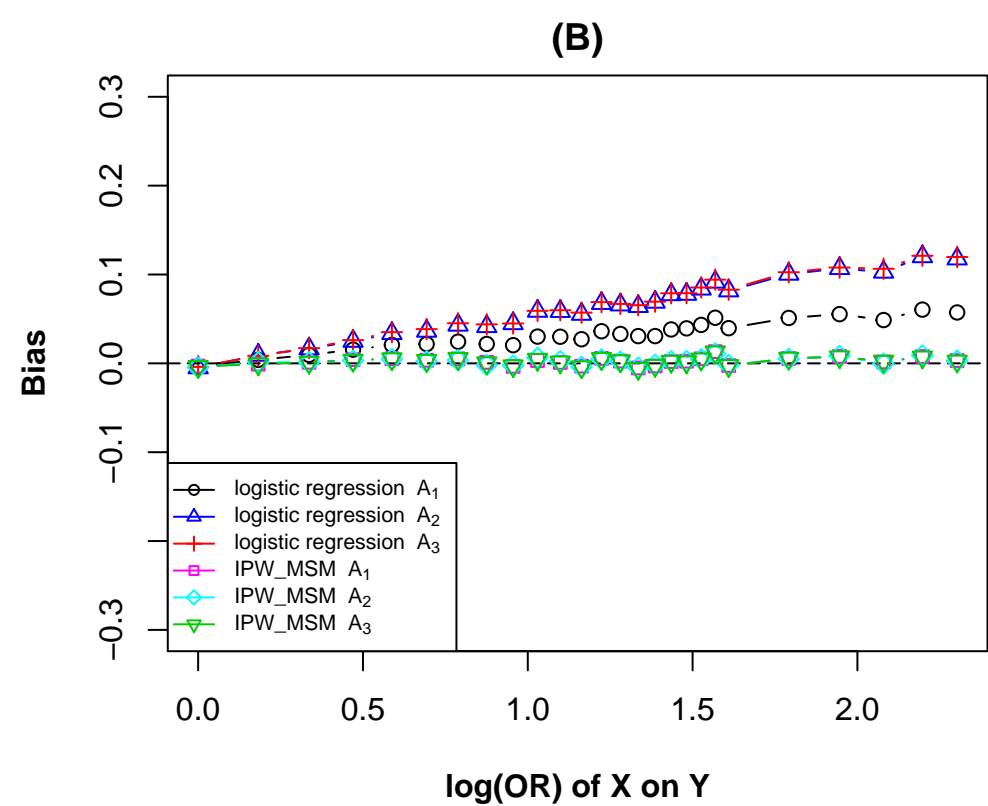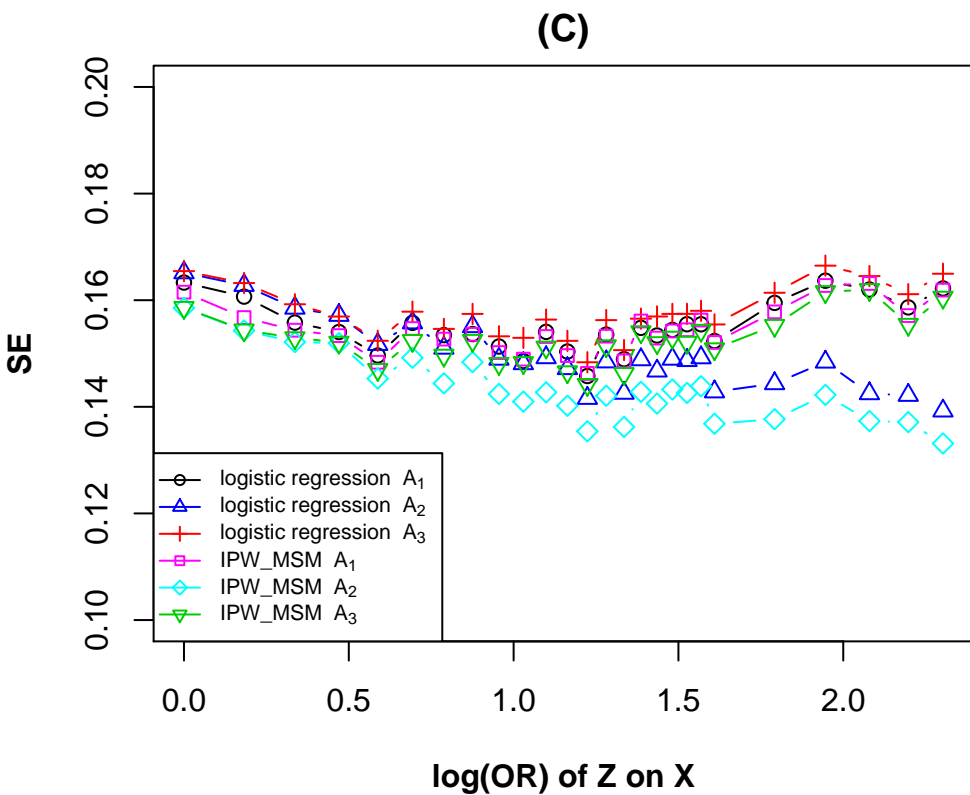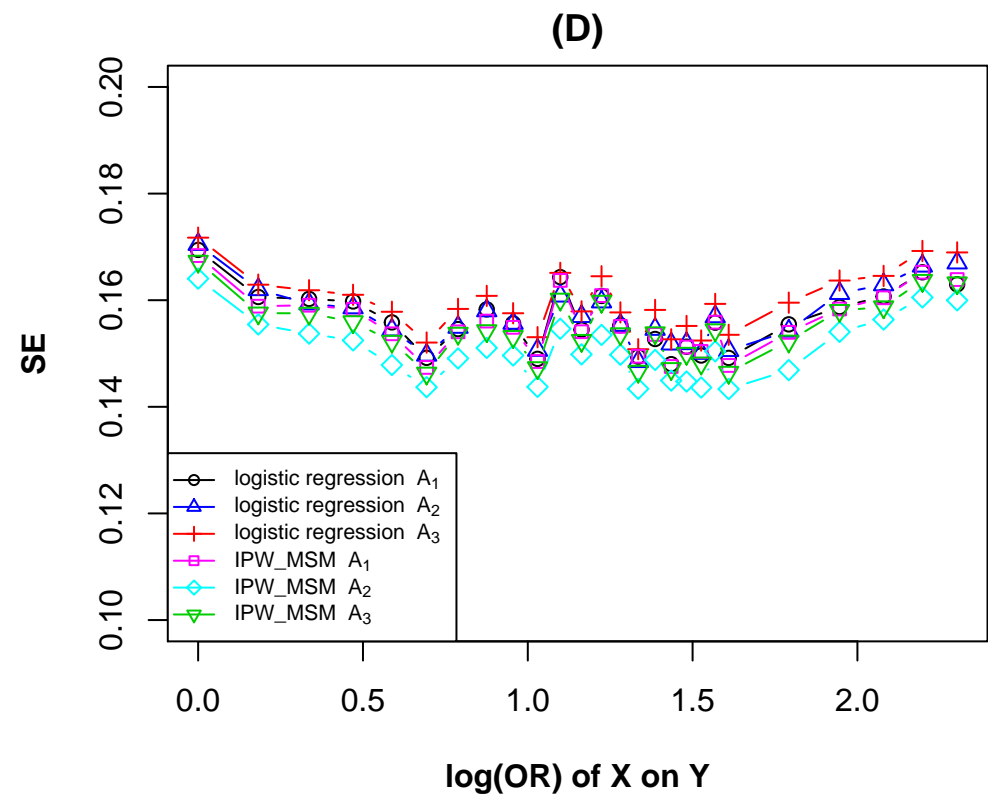

Supplement: Supplementary file 11 — Scenario 4 (Figure 1d), simulation results of the bias and standard error of c-equivalence sets A 1 ≈ A 2 ≈ A 3 when varied across the log transformed odds ratio effect of Z on X and X on Y. (PDF 25 kb) [file 12874_2017_449_MOESM11_ESM.pdf]

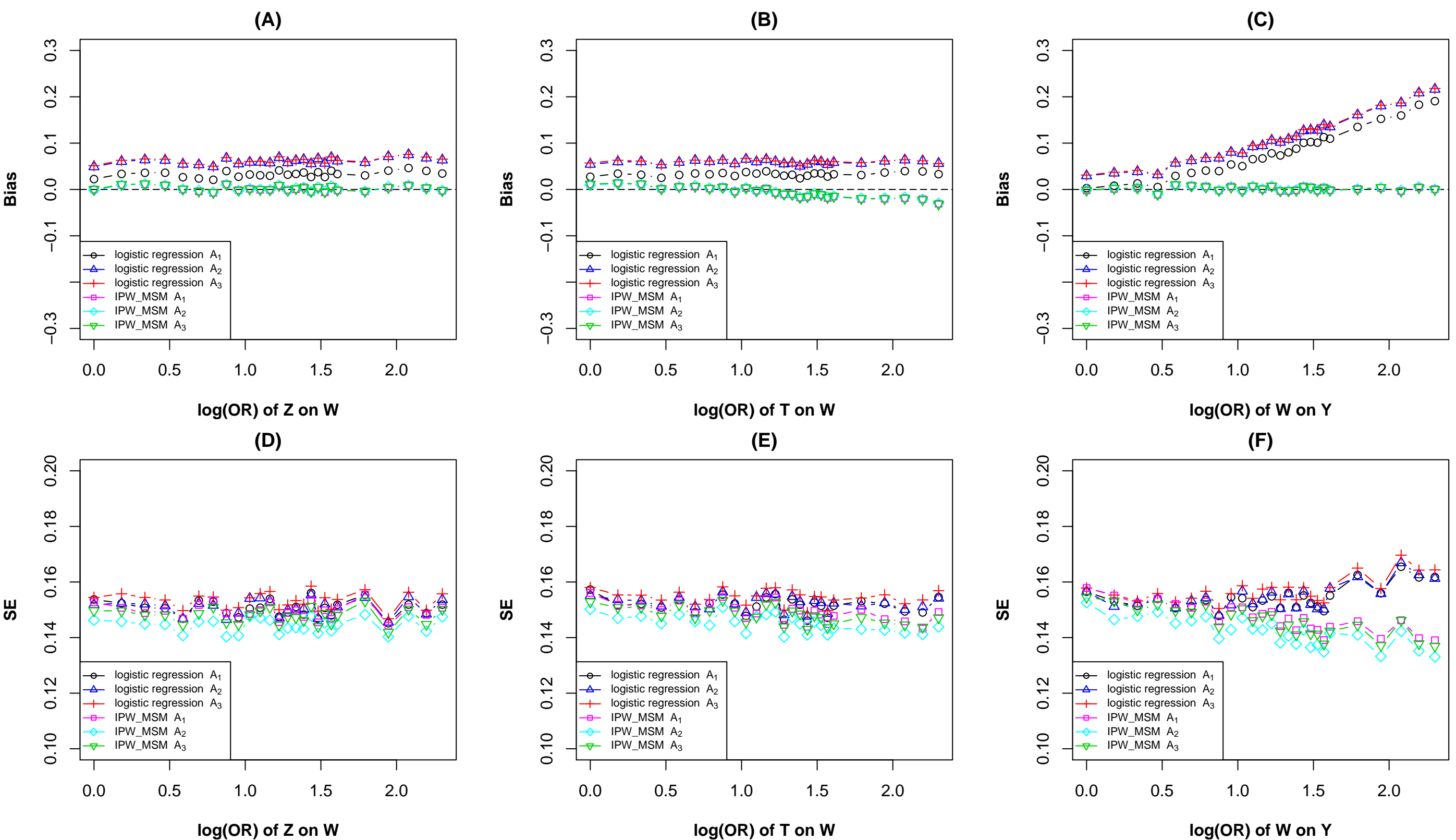

Supplement: Supplementary file 12 — Scenario 4 (Fig. 1d), simulation results of the bias and standard error of c-equivalence sets A 1 ≈ A 2 ≈ A 3 when varied across the log transformed odds ratio effect of Z on W,T on W and W on Y. (PDF 35 kb) [file 12874_2017_449_MOESM12_ESM.pdf]
